# Mutations in single *FT*- and *TFL1*-paralogs of rapeseed (*Brassica napus* L.) and their impact on flowering time and yield components

**DOI:** 10.3389/fpls.2014.00282

**Published:** 2014-06-17

**Authors:** Yuan Guo, Harloff Hans, Jung Christian, Carlos Molina

**Affiliations:** Plant Breeding Institute, University of KielKiel, Germany

**Keywords:** *Flowering Locus-T*, *Terminal Flower-1*, TILLING, rapeseed, allopolyploid, differential function

## Abstract

Rapeseed (*Brassica napus* L.) is grown in different geographical regions of the world. It is adapted to different environments by modification of flowering time and requirement for cold. A broad variation exists from very early-flowering spring-type to late-flowering winter cultivars which only flower after exposure to an extended cold period. *B. napus* is an allopolyploid species which resulted from the hybridization between *B. rapa* and *B. oleracea*. In *Arabidopsis thaliana*, the PEBP-domain genes *FLOWERING LOCUS-T (FT)* and *TERMINAL FLOWER-1 (TFL1)* are important integrators of different flowering pathways. Six *FT* and four *TFL1* paralogs have been identified in *B. napus*. However, their role in flowering time control is unknown. We identified EMS mutants of the *B. napus* winter-type inbreed line Express 617. In total, 103 mutant alleles have been determined for *BnC6FTb, BnC6FTa*, and *BnTFL1-2* paralogs. We chose three non-sense and 15 missense mutant lines (M_3_) which were grown in the greenhouse. Although only two out of 6 *FT* paralogs were mutated, 6 out of 8 *BnC6FTb* mutant lines flowered later as the control, whereas all five *BnC6FTa* mutant lines started flowering as the non-mutated parent. Mutations within the *BnTFL1-2* paralog had no large effects on flowering time but on yield components. F_1_ hybrids between *BnTFL1-2* mutants and non-mutated parents had increased seed number per pod and total seeds per plant suggesting that heterozygous mutations in a *TFL1* paralog may impact heterosis in rapeseed. We demonstrate that single point-mutations *in BnFT* and *BnTFL1* paralogs have effects on flowering time despite the redundancy of the rapeseed genome. Moreover, our results suggest pleiotropic effects of *BnTFL1* paralogs beyond the regulation of flowering time.

## Introduction

Rapeseed (*Brassica napus* L.) is a major oil crop accounting for more than 60 million tons of seed and more than 20 million tons of extracted oil worldwide (http://www.worldoil.com/). This crop is widely cultivated in several temperate regions of the world such as northern Europe, Canada, China and Australia. Adapting flowering time to regional environmental conditions has been a major target of rapeseed breeding. A broad variation exists from very early-flowering spring-type to late-flowering winter cultivars that only flower after exposure to an extended cold period, a process known as vernalization (Iniguez-Luy and Federico, 2011). In rapeseed, flowering time and yield are closely linked to each other. Several genomic regions have been reported to contain major QTL for both traits. In a doubled haploid (DH) population derived from a cross between the Chinese semi-winter line Ningyou7 and the European winter-type Tapidor, at least four flowering time QTL were suggested as indicator QTL for yield (Long et al., 2007; Shi et al., 2009). Genetic variation within the different rapeseed types is relatively small, implying a need for wide crosses between non-adapted ecotypes to introduce traits of interest into elite materials (Girke et al., 2012). However, the introgression of genes from non-adapted ecotypes into elite cultivars is difficult due non-adapted flowering time. Therefore, it is of great interest to measure the effects of different paralogs on flowering and other characters.

*Brassica napus* and its close relatives *Brassica oleracea* and *Brassica rapa* belong to the family Brassicaceae which also includes the model plant *Arabidopsis thaliana*. Rapeseed is an allotetraploid species originating from the spontaneous hybridization between *B. rapa* (AA) and *B. oleracea* (CC) less than 5000 years ago (Ziolkowski et al., 2006; Wang et al., 2011). These two progenitor species are ancient polyploids that underwent genome triplication between the *Brassica-Arabidopsis* split (~13 MYA) and their actual divergence event (~two MYA). Comparative mapping between the *Arabidopsis* and *Brassica* genomes revealed numerous homologous regions arranged in highly syntenic chromosome blocks. Many Arabidopsis genes are represented in the *B. napus* genome by at least three paralogs (Schranz et al., 2006). Due to their close phylogenetic relationship and the high economic importance of rapeseed, knowledge transfer from the model species Arabidopsis to the complex Brassica genomes constitutes a worthwhile challenge for genomics research.

In Arabidopsis, four pathways controlling flowering time have been extensively studied (Amasino, 2010). All these pathways converge at the *CO/FT* regulon (Andres and Coupland, 2012). Under long day (LD) conditions, the CONSTANS (CO) protein accumulates in leaves and induces expression of the floral integrator gene *FLOWERING LOCUS T (FT)* in the phloem companion cells (Moon et al., 2003; Turck et al., 2008). FT is the long-sought “florigen” and it is reported to be a strong mobile signal triggering activation of floral identity genes in the Arabidopsis shoot apical meristem (Andres and Coupland, 2012). The FT protein is transported via the sieve tubes to the shoot apex, where it forms a heterodimer with the FD (FLOWERING LOCUS D) protein (Abe et al., 2005; Wigge et al., 2005). Interestingly, a very closely related gene, *TERMINAL FLOWER 1* (*TFL1*) plays an *FT*-antagonistic role by competing for FD, leading to a repression of floral transition (Valverde, 2011; Andres and Coupland, 2012). In Arabidopsis, *FT*-overexpressing plants and *TFL1* non-sense mutants show the same early-flowering phenotype and produce terminal flowers in the shoot apex. *TFL1* represses transcription of genes which are activated by *FT* (Hanano and Goto, 2011). In Arabidopsis, these two highly similar polypeptides belong to a family of six members characterized by the phosphatidylethanolamine-binding domain (PEBP) (Kardailsky et al., 1999). Substitutions of crucial amino acids from the *FT* and *TFL1* exon II, as well as the exchange of the exon IV led to contrasting protein functions for both polypeptides (Hanzawa et al., 2005; Ahn et al., 2006).

Apart from their major role to control flowering time, *FT* and *TFL1* orthologs have been shown to alter a variety of phenotypic characters. In tomato, the *SINGLE FLOWER TRUSS* (*FT* ortholog) and *SELF PRUNING* (*TFL1* ortholog) genes impact fruit yield heterosis. F_1_ hybrids generated by crosses between loss of function *SFT* mutants and tomato wild type (WT) plants of the non-mutagenized line M82 have shown strong increment in fruit production (Molinero-Rosales et al., 2004; Krieger et al., 2010). *FT/TFL1* gene orthologs have been characterized in diploid crops such as rice (Kojima et al., 2002), pea (Hecht et al., 2011), barley (Faure et al., 2007), poplar (Böhlenius et al., 2006), and sugar beet (Pin et al., 2010) and in two polyploids, wheat (Yan et al., 2006) and potato (Navarro et al., 2011). The characterization of the *FT/TFL1* gene orthologs in polyploid plants is a special challenge because duplicated genes can build new regulation networks leading to sub- or neo-functionalization (Pin and Nilsson, 2012).

Rapeseed has six *BnFT* paralogs (*BnA2FT, BnC2FT, BnC6FTa, BnC6FTb, BnA7FTa*, and *BnC7FTb*) sharing high sequence similarity (92–99%) in their four exons (Wang et al., 2009). It has been shown that the *BnC2FT* copy is silenced in *B. napus* and *B. oleracea* due to the insertion of a miniature inverted-repeat transposable element (MITE) in its promoter region, whereas the remaining five copies are detectable in *B. napus, B. rapa* and *B. oleracea* (Wang et al., 2012a). There are at least four *TFL1* paralogs in the *B. napus* genome (Mimida et al., 1999). Among them, the *BnTFL1-2* paralog shares high homology with the *B. rapa* ortholog on chromosome A10, whereas *BnTFL1-1, BnTFL1-3* are highly similar to their *B. oleracea* counterparts. The *BnC6FTa* and *BnC6FTb* paralogs were co-located to a major flowering time QTL detected in nine winter-cropped environments which could support their function as flowering time regulators in *B. napus* (Qiu et al., 2006; Shi et al., 2009). Until now, *B. napus FT/TFL1* homologs have not been functionally characterized.

This study had two major aims. First, we aimed to uncover the role in flowering time control of different *FT* and *TFL1* paralogs in *B. napus* by analyzing EMS-treated offspring with missense and splice-site mutations within selected paralogs. We demonstrate that single mutations can change the onset of flowering in *B. napus* despite the redundancy of its allopolyploid genome. Moreover, we postulated that *BnTFL1* mutations also affect seed yield components in rapeseed. We found increased seed yield in F_1_ plants carrying a mutated *BnTFL1* allele on the Express 617 background. Our data suggest that EMS-generated alleles may constitute a new resource to broaden the genetic basis of rapeseed breeding.

## Materials and methods

### Plant materials and greenhouse experiments

Seedlings of M_3_ lines and Express 617 (controls) were grown in the greenhouse at constant temperature (22°C) under long days (LD, 16 h light/8 h dark) for 4 weeks. Express 617 is an inbreed line (F_11_) originated from the rapeseed winter-type cultivar Express (Harloff et al., 2012). Subsequently, plants were vernalized for 8 weeks at 4°C under LD conditions in a cold chamber. Of each M_3_ line, 30 plants were grown. After vernalization, plants were returned to the initial greenhouse conditions and transplanted to 11 × 11 cm pots. M_3_ plants and Express 617 controls were arranged in randomized blocks. Plant positions on the greenhouse were indexed and linked to randomly generated numbers using the Microsoft Excel software. Selected M_3_ lines were crossed with the male sterile (MS) line MSL007 (NPZ, Hohenlieth, Germany) using homozygous M_3_ plants as a pollinators. F_1_ plants and Express 617 controls were grown in the greenhouse under the conditions mentioned above. F_2_ populations were produced by crossing M_3_ homozygous plants from a selected *BnC6FTb* mutation (*BnC6FTb*_*G*2154*A*_) and non-mutagenized Express 617 plants. In each greenhouse experiment, the following phenotypic characters were measured according to the BBCH scale (http://www.jki.bund.de/en/startseite/veroeffentlichungen/bbch-codes.html): first non-cotyledonal leaves (NCL, BBCH10), rosette plant (BBCH30), visible floral buds (BBCH50), first open flower (BBCH60), and end of flowering (BBCH69). Plants that did not grow beyond NCL (BBCH 10) were excluded from the experiment. Plant height, number of branches, initial flowers, filled pods, seed number and seed weight were recorded for each plant separately.

### Mutation screening

A total of 3488 M_2_ plants of the Express 617 EMS-population were screened by TILLING as described by Harloff et al. (2012). Gene specific primers were designed for *BnC6FTa* (FJ848915.1), *BnC6FTb* (FJ848917.1), and *BnTFL1-2* (AB017526.1) (Supplementary Table S1). For primer design and comparative analysis, *B. rapa* and *B. oleracea* genome sequences were downloaded from (http://brassicadb.org/brad/downloadOverview.php) and (http://ocri-genomics.org/bolbase/), respectively. Plant genomic DNA arrayed in two dimensional 8-fold pools was amplified by direct or nested PCR. Forward and reverse primers were 5′-end labeled with 700 nm (DY-681) or 800 nm (DY-781) IRD fluorescence dyes, respectively (Biomers, Ulm, Germany, www.biomers.net). PCR amplifications with labeled oligos were done using the following profile: 95°C 5 min; 35 cycles of 95°C 30 s, 60°C 45 s, 72°C 90 s, 72°C 10 min. Heteroduplex-specific restriction endonuclease *CEL1* was extracted from celery and stored at −80°C as reported by Frerichmann et al. (2013). Labeled fragments were separated by a LI-COR 4300 DNA analyzer (LI-COR Biosciences) for 3:15 to 4:15 h at 1,500 V, 40 mA and 40 W. Gel images were analyzed using the software GelBuddy (http://www.proweb.org/gelbuddy/). After positive pools had been identified, single plant DNA was amplified with unlabeled oligos and sequenced for SNPs confirmation. Sequences were analyzed with the CLC-bio Main Workbench sequence alignment tool (CLC bio, Aarhus, Denmark).

### DNA isolation and genotyping

Total DNA was extracted from young leaves using a CTAB protocol (Morjane et al., 1994). Total DNA was treated with RNAse I (Fermentas, www.fermentas.de), and DNA concentration was determined by spectrometry (NanoDrop, www.nanodrop.com). DNA quality was checked by 1% agarose gel electrophoresis. For genotyping mutant lines, genomic DNA from single plants was amplified by PCR using unlabeled primers. PCR was done essentially as described in the previous paragraph. Five micro litter of each PCR product were loaded on 1% (w/v) agarose gels. Upon band size confirmation, the remaining 25 μl of PCR product were sequenced via Sanger capillary sequencing. The sequences were analyzed with the CLC-Bio software (CLC bio, Aarhus, Denmark) using the sequence assembly viewer tool.

### Tissues sampling and RT-qPCR

Young leaves of M_3_ plants and Express 617 controls were sampled at four developmental stages, as described above. Genomic DNA sequences from the different flowering time genes analyzed were retrieved from the non-redundant NCBI nucleotide database (http://www.ncbi.nlm.nih.gov/). Individual sequences were loaded to the CLC-bio main workbench version 6.0 (http://www.clcbio.com), and groups of paralogs were aligned with the help of the internal alignment routine. Two main strategies were applied for expression analysis: (i) primers were designed in conserved regions within groups of paralogs for detecting joint gene expression, and (ii) copy-specific primers were designed for the members of selected paralog genes (Supplementary Table S1). Total RNA was extracted using the RNeasy kit (QIAGEN, www.qiagen.com) according to the manufacturer's protocol. The RNA concentration was determined by spectrometry (Nano Drop; Thermo Scientific, Wilmington, USA) and quality was checked by agarose gel electrophoresis. Total RNA was treated with DNAse I (Fermentas Inc., Maryland, USA). First-strand cDNA was synthesized using Oligo(dT)_18_ primers and the M-MuLV Reverse Transcriptase (Fermentas).

Quantitative real-time RT-PCR (RT-qPCR) was performed with SYBR qPCR Super mix w/ROX (Invitrogen Corporation, Carlsbad, USA) using a CFX96 Real-Time System (Bio-Rad Laboratories GmbH, München, Germany). Reactions were performed in a total volume of 15 μ l containing 100 nM of each primer and 2 μ l of diluted cDNA templates, and amplified using the following cycling conditions: 95°C for 3 min, 40 cycles of 95°C for 10 s, 60°C for 30 s, and 72°C for 30 s, followed by 95°C for 10 min. A melting curve was generated using a temperature range from 65°C to 95°C with increments of 0.5°C every 5 s. For each sample at least three technical replications were performed. For data analysis, the mean C_*t*_ value of the target gene was normalized against the average C_*t*_ value of two housekeeping genes (*BnGADPH-3* and *BnB-Tub*). Calculation of relative expression values was carried out following Pfaffl (2001) after extracting main Ct values via CFX manager software (Bio-Rad Laboratories GmbH, München, Germany). In each analysis, the relative expression value for the reference sample has been set to 1. Normalized expression was averaged over two biological replicates and three technical repetitions in each case. Standard curves for the target and housekeeping genes are based on dilution series of purified cloned fragments for each gene.

### Sequence diversity analysis

For analyzing sequence diversity within the *BnC6FTb* and *BnTFL1-2* genes, genomic DNA from one-hundred accessions of the *B. napus* ASSYST panel was amplified with paralog-specific primers and sequenced via Sanger method. We selected 117 lines from the *B. napus* ASSYST diversity set (Bus et al., 2011) including winter, semi-winter and spring types which had been phenotyped in several environments worldwide (Supplementary Table S2). Lyophilized leaf samples harvested from young plants were used for DNA isolation with the NucleoSpin Plant II DNA isolation kit (Macherei & Nagel, Germany), following the manufacturer's instructions. PCR amplifications were carried out with paralog-specific primers as follows: 95°C 5 min; 35 cycles of 95°C 30 s, 60°C 45 s, 72°C 90 s, 72°C 10 min. Sequences resulting from single band amplicons were assembled and aligned using the CLC-bio main workbench software (CLC bio, Aarhus, Denmark) and the resulting FASTA alignment was loaded into the software TASSEL (http://www.maizegenetics.net) for identification of polymorphic SNPs.

## Results

### Paralog-specific expression of five *BnFT* genes

**Figure 1:**
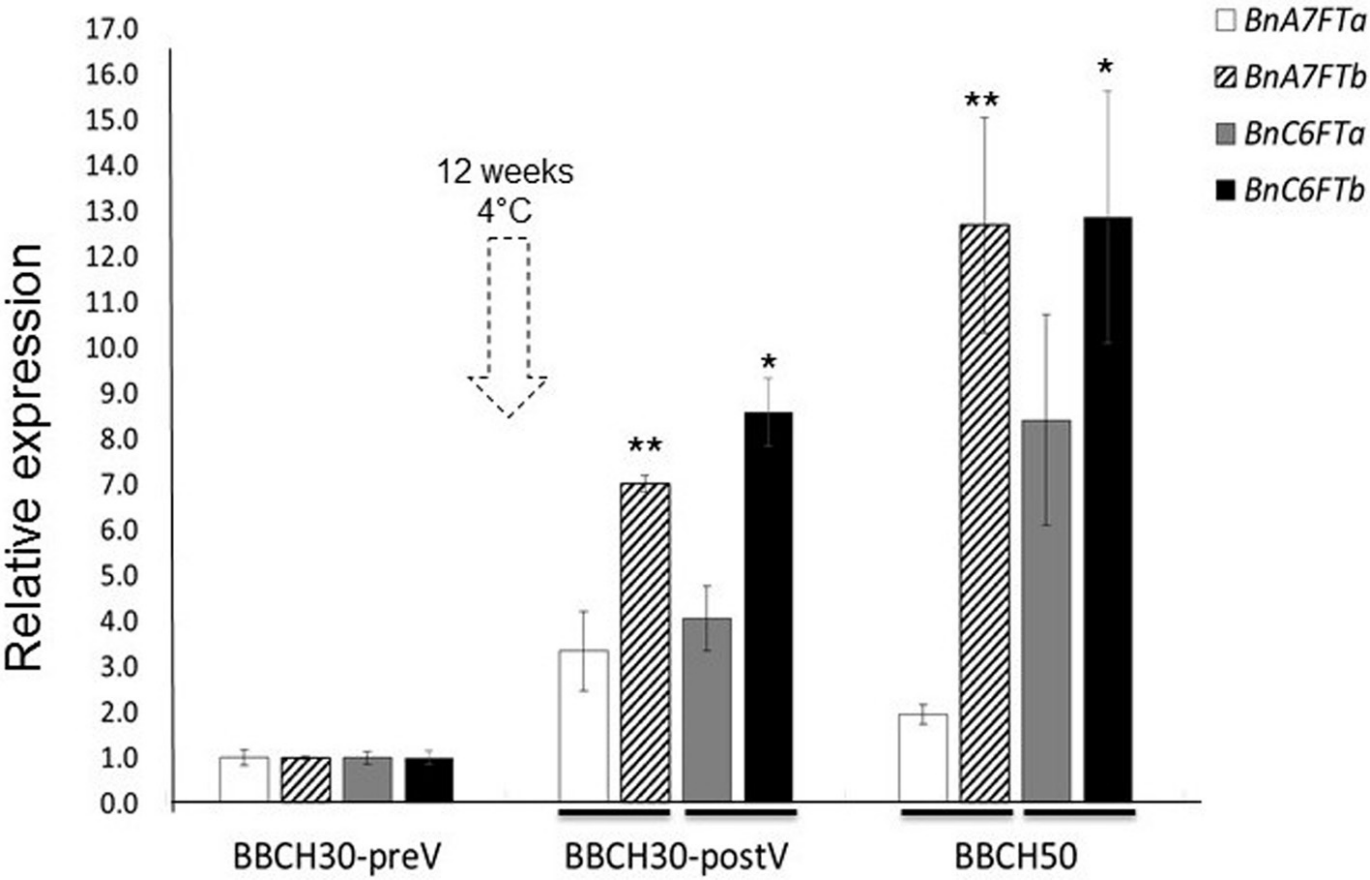
**Relative expression of five *BnFT* paralogs in Express 617 plants at three developmental stages before and after vernalization (dotted arrow)**. Plants at BBCH30 were analyzed before vernalization (preV) and after vernalization (postV). The time point BBCH30-preV was taken as reference for calculation of relative expression in all target genes. Two biological replicates and three technical repetitions were analyzed for each time point. Error bars indicate the standard error of the mean of the relative expression values. Expression levels of target *BnFT* genes were normalized against *BnGAPDH* total expression. Ct values of the paralogs *BnA2FT* and *BnC2FT* were below the detection threshold at BBCH30 and BBCH50. Significant differences (*P* < 0.05) are depicted by asterisks. Differences in relative expression between the *BnC6FTa*/*BnC6FTb* (^*^) and *BnA7FTa*/*BnA7FTb* paralogs (^**^) at each time point were tested via *t*-test. Lines at the base of the bars indicate the comparison pairs. All samples were taken between zeitgeber 11 h and 12 h in each developmental stage. Express 617 plants reached BBCH30-preV ~30 days after sowing. BBCH30-postV was registered ~90 days after sowing. BBCH50 was registered ~107 days after sowing.

We carried out a RT-qPCR experiment to measure the paralog specific expression of six *BnFT* paralogs in leaves of the winter-type inbred line Express 617 during the transition to reproductive stages. Samples were taken from greenhouse-grown plants at three different stages of development (BBCH30 before and after vernalization and BBCH50). Relative expression values for each paralog were calculated after Ct normalization using *BnGAPDH* as a reference gene. Leaf samples at BBCH30 before vernalization (preV) were used as reference samples for relative expression calculations. At BBCH30 before vernalization (BBCH30-preV), four *BnFT* paralogs (*BnC6FTa/b* and *BnC6FT7a/b*) were weakly expressed (Figure 1), whereas two transcripts were not expressed (*BnC2FT* and *BnA2FT)*. Moreover, *BnA2FT* was only highly expressed at BBCH60 after floral transition (data not shown), whereas *BnC2FT* showed no expression at all. After vernalization (BBCH30-postV), *BnC6FTa/b* and *BnA7FTa/b* expression was higher in rosette plants, but differences between paralogs were obvious. *BnC6FTb* showed the largest relative expression level (~9-fold). At BBCH50 (visible floral buds), the *BnC6FTb* and *BnA7FTb* paralogs showed the largest relative expression levels (~13-fold) (Figure 1). In leaves at BBCH 60 (first flower open), all paralogs with exception of *BnC2FT* showed very high relative expression levels (>2000-fold), where *BnC6FTa* showed the highest relative expression (data not shown).

### Ems mutations in *BnFT* and *BnTFL1* paralogs

We screened our EMS population to measure the flowering time effect of mutations within the *BnFT* paralogs *BnC6FTa* (FJ848915.1) and *BnC6FTb* (FJ848917.1). In Arabidopsis, apart from FT, other PEBP proteins such as TERMINAL FLOWER-1 (TFL1) regulate flowering by competing with FT for its binding targets (Mimida et al., 2001). Therefore, we developed primers for the *BnTFL1-2* (ABO17526) gene assigned to *B. rapa* chromosome A10. In total, 3488 M_2_ plants were screened by TILLING for EMS-induced mutations in *BnC6FTa/b* and *BnTFL1-2*.

**Figure 2:**
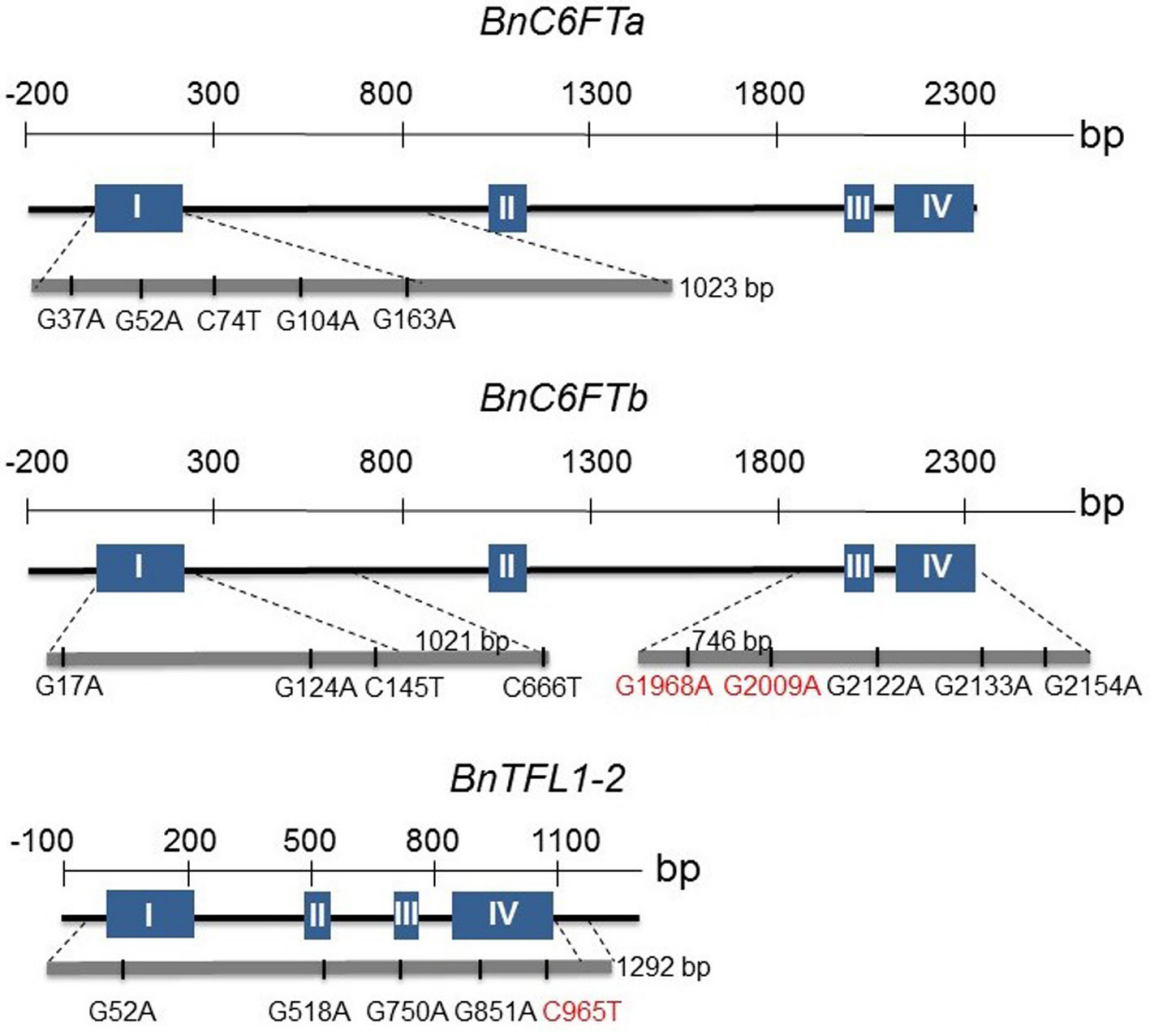
**Graphical presentation of 18 point mutations in two *FT* (*BnC6FTa, BnC6FTb*) and one *TFL1* (*BnTFL1-2)* paralog of the *B. napus* winter-type cultivar Express 617**. The exon (blue boxes) - intron (black lines) structure of each gene is shown. Locations of SNPs are depicted by the gray bar below. Numbers refer to the position of the mutations from the START codon. Only STOP/splice-mutations (red) and selected missense mutations are shown.

We generated paralog-specific PCR amplicons covering between 50% (*BnC6FTa*) and 100% (*BnC6FTb* and *BnTFL1-2*) of the open reading frames. The *BnC6FTa* fragment covered exon I and intron I. The two *BnC6FTb* fragments covered exon I / intron I and exons III / IV (Figure 2). The *BnTFL1-2* fragment covered all four exons. We identified 55, 14, and 34 single nucleotide mutations in the *BnC6FTb, BnC6FTa*, and *BnTFL1-2* genes, respectively. Forty-three mutations are located in introns, 19 are silent mutations, and three are located within the UTRs (Table 1). Mutation rates ranged between 1/72 kb and 1/24 kb per 1000 plants. The names of the mutant alleles contain the nucleotide substitution and nucleotide position (Table 2).

**Table 1:** **EMS mutations in three flowering time genes detected by TILLING of the winter-type inbred line Express 617**.

	***BnC6FTa***	***BnC6FTb***	***BnTFL1-2***
Number of paralogs in rapeseed	6	6	4
Total number of M_2_ plants screened	3488	3488	2092
Sequence screened by TILLING (bp)	1023	1767	1292
Nonsense mutations	0	1	2
Missense mutations	6	15	10
Splice site mutations	0	1	1
Total number of mutations	14	55	34
Mutations/kb	72	30	24
M_3_ families selected for phenotyping	5	9	5
Total number of mutations	103		

The BnC6FTb paralog was screened by two different fragments, 1021 bp and 746 bp in size.

**Table 2:** **Nucleotide position and amino acid changes due to EMS mutations in 18 missense/non-sense mutations in three *B. napus* flowering time regulators**.

**Gene**	**Mutation**	**Exon**	**Amino acid substitution**	**Mutant code**
*BnC6FTa*	G37A	Exon I	Gly13Arg	*C6FTa*_*G*37*A*_
	G52A	Exon I	Val17Lle	*C6FTa*_*G*52*A*_
	C74T	Exon I	Ser25Leu	*C6FTa*_*C*74*T*_
	G104A	Exon I	Arg35lys	*C6FTa*_*G*104*A*_
	G163A	Exon I	Glu55Lys	*C6FTa*_*G*163*A*_
*BnC6FTb*	G17A	Exon I	Arg6Lys	*C6FTb*_*G*17*A*_
	G124A	Exon I	Asp42Asn	*C6FTb*_*G*124*A*_
	C666T	CArG Box	CArG Box	*C6FTb*_*C*666*T*_
	G1968A	Exon III	Trp88Stop	*C6FTb*_*G*1968*A*_
	G2009A	Intron III	Splice site	*C6FTb*_*G*2009*A*_
	G2122A	Exon IV	Arg112Lys	*C6FTb*_*G*2122*A*_
	G2133A	Exon IV	Gly116Arg	*C6FTb*_*C*2133*T*_
	G2154A	Exon IV	Val123Met	*C6FTb*_*G*2154*A*_
*BnTFL1-2*	G52A	Exon I	Val18Lle	*TFL1-2*_*G*52*A*_
	C518T	Exon II	Pro83Ser	*TFL1-2*_*C*518*T*_
	G750A	Exon III	Gly105Arg	*TFL1-2*_*G*750*A*_
	G851A	Exon IV	Val108Met	*TFL1-2*_*G*851*A*_
	C965T	Exon IV	Gln146Stop	*TFL1-2*_*C*965*T*_

We identified one non-sense mutation in exon III of the *BnC6FTb* gene (*BnC6FTb*_*G*1968*A*_) leading to a truncated protein by substitution of a tryptophan by a stop codon (position 88). Another mutation (*BnC6FTb*_*G*2009*A*_) resulted in a splice-site deletion leading to a truncated protein by interrupting the junction between exons III and IV. The *BnTFL1-2*_*C*965*T*_ mutation in Exon III results in the substitution of a glutamine by a stop codon (position 146). Moreover, we detected numerous missense mutations in *BnC6FTa* (15), *BnC6FTb (16)*, and *BnTFL1-2* (10).

We decided to focus on splice site- and missense-mutations which are most likely to affect the protein function. All observed missense mutations were compared to the SIFT database (http://sift.jcvi.org) in order to evaluate the impact of the amino acid substitutions on the protein function (data not shown). According to this analysis, we selected 18 M_3_ lines for growth experiments in the greenhouse (5 *BnC6FTa*, 8 *BnC6FTb*, and 5 *BnTFL1-2* mutations) (Supplementary Table S3) (Figure 2).

### Phenotypic characterization of *BnFT* and *BnTFL1-2* mutants

**Figure 3:**
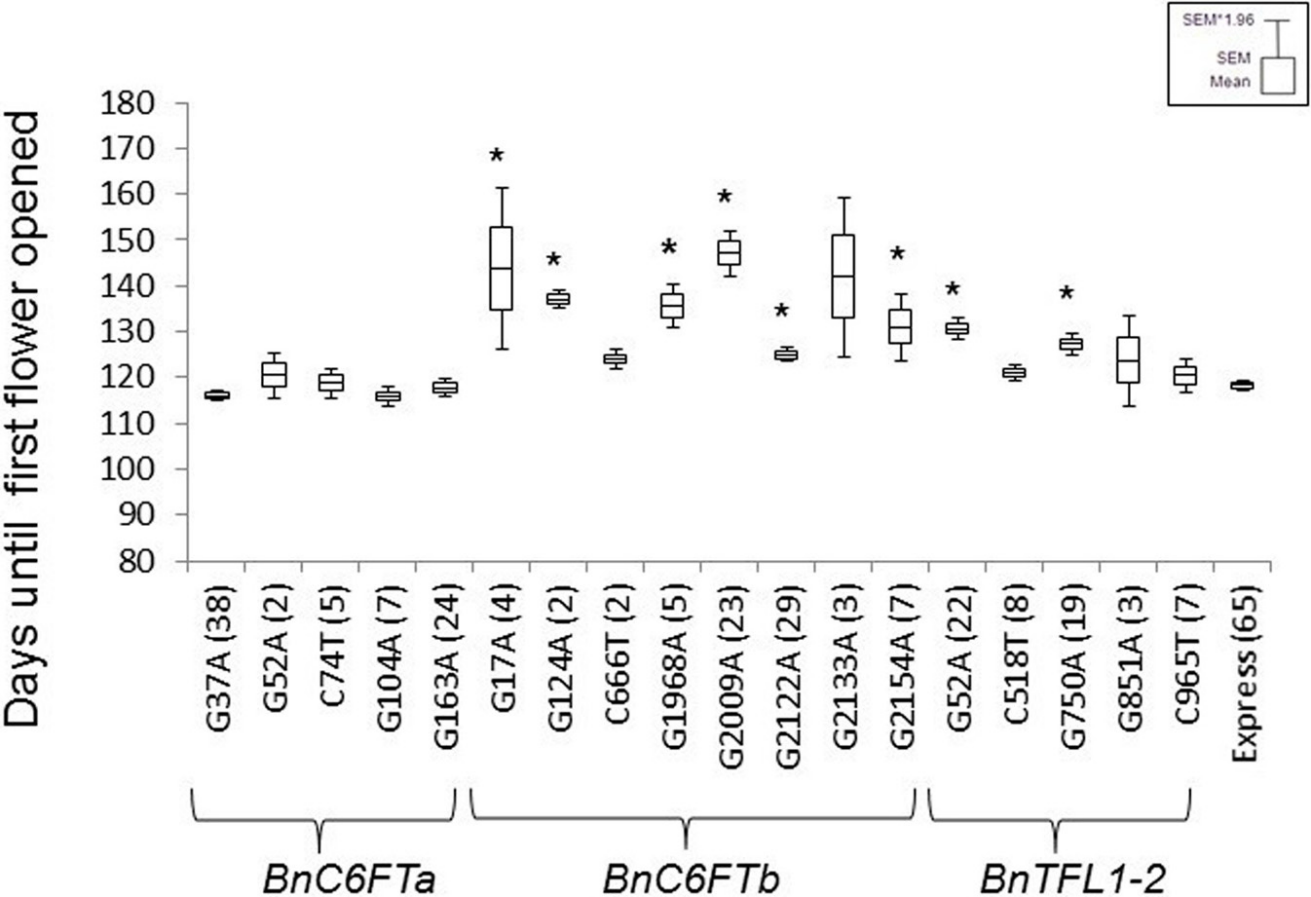
**Flowering time point of 18 *BnC6FT*- and *BnTFL1-2* mutants grown in the greenhouse at constant temperature (22°C), and LD (16 h light) after vernalization (4°C, 16 h light, 8 weeks)**. Days to flowering (BBCH 60) was measured in M_3_ plants homozygous for the EMS allele. The non-mutagenized donor line Express 617 was used as a control. The number of plants analyzed is written in brackets. Differences in flowering time between homozygous mutants and control plants were tested via *t*-test. Significant differences (*P* < 0.05) are depicted by asterisks.

**Figure 4:**
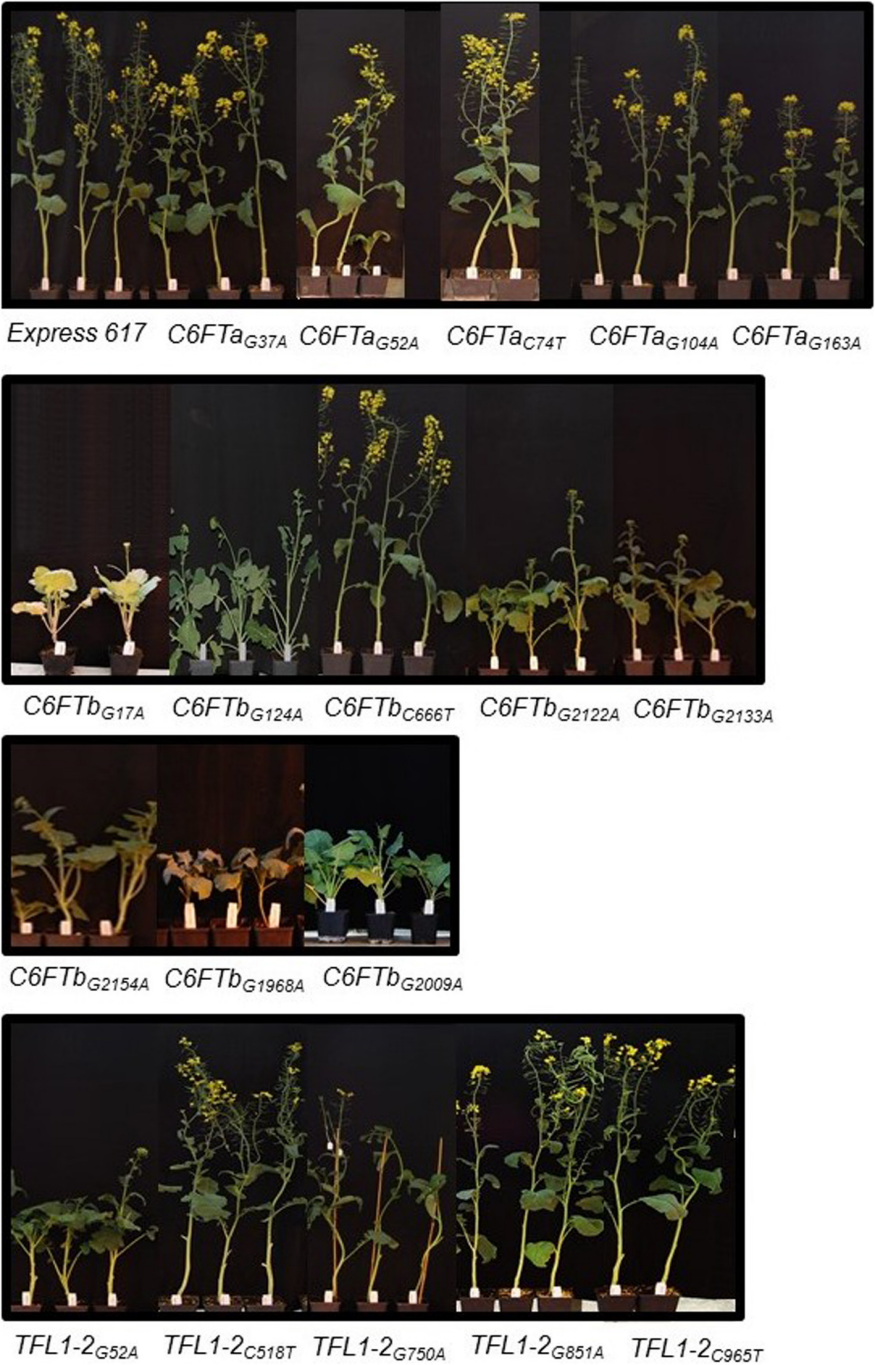
**Phenotypes of 18 *B. napus BnC6FTa/b* and *BnTFL1-2* EMS M_3_ lines**. Photos were taken as the non-mutagenized Express 617 plants started flowering (top left). Plants were grown in the greenhouse at constant temperature (22°C), and LD (16 h light) after vernalization (4°C, 16 h light, 8 weeks).

First, we confirmed the genotype of each selected M_2_ plant by Sanger sequencing. Then, M_3_ lines were grown in the greenhouse together with non-mutagenized Express 617 plants as a control. The phenological development of *BnC6FTa* and *BnC6FTb* lines was clearly different. All five *BnC6FTa* mutants flowered as the control, whereas six out of eight *BnC6FTb* mutants flowered later (Figure 3). The *C6FTb*_*G*1968*A*_ mutants (stop mutation) showed a flowering delay of ca. 18 days, while *C6FTb*_*G*2009*A*_ splice-site mutants flowered 29 days later as the control. Interestingly, 40 and 54% of the *C6FTb*_*G*1968*A*_ and *C6FTb*_*G*2009*A*_ M_3_ mutants, respectively, did not bolt at all (Figure 4). *BnC6FTb* missense mutants started flowering 7 days (*BnC6FTb*_*C*2122*A*_) up to 26 days (*BnC6FTb*_*G*17*A*_) later as the control. Apart from flowering time, reduced fertility was also apparent, mostly in *BnC6FTb* M_3_ plants (Supplementary Figure 1).

To evaluate the effect of background mutations on flowering time, we produced an F_2_ population by crossing *BnC6FTb*_*G*2154*A*_ M_3_ plants with non-mutagenized Express 617. *BnC6FTb*_*G*2154*A*_ M_3_ missense mutants gave higher hybrid seed yield as the stop mutants and they flowered 15 days later as the control. *BnC6FTb*_*G*2154*A*_ M_3_ plants showed a reduced number of initial flowers in comparison to other M_3_ mutants, however most flowers were fertile. A total of 26 F_2_ plants encompassing all three genotypic classes were grown in the greenhouse together with Express 617. In agreement with M_3_ observations, homozygous F_2_ mutants (*ft ft*) flowered 13 days later than F_2_ siblings homozygous for the wild-type allele (*FT FT*) which did not show any significant differences in flowering time as compared to non-mutagenized Express 617 (Supplementary Figure 2).

The stop mutation *BnTFL1-2*_*C*965*T*_ did not lead to a major delay in flowering time. In contrast, the missense mutants *BnTFL1-2*_*G*52*A*_ and *BnTFL1-2*_*G*750*A*_ flowered ~10 days later than the control (Figure 3). Since, the stop mutation is close to the end of the *BnTFL1-2* gene, a functional protein may still arise after translation. Furthermore, *BnTFL1*_*G*750*A*_ mutants exhibited modifications in plant architecture which gave us a reason to select them for crossing experiments.*BnTFL1*_*G*750*A*_ mutants developed normally during the early growth phase until reaching BBCH50 (visible floral buds). The internode elongation phase was much longer as compared to Express 617, as a consequence, mutant plants were not able to stand by themselves after BBCH50. In this M_3_ line, the flower development limited the continuous growth of the floral meristem, whereas side branches continued flowering.

### The EMS mutations in *BnC6FTb* and *BnTFL1-2* paralogs are located in highly monomorphic regions of exon III and exon IV

To investigate the genetic structure of those *BnFT* and *BnTFL1* paralogs with paramount impact on flowering time, we analyzed the sequence diversity of *BnC6FTb* and *BnTFL1-2* in *B. napus* by sequencing their complete exons III and IV in 117 *B. napus* inbreed lines from different geographic origins and growth types. Sequences selected for analysis in each gene after quality trimming are deposited in Supplementary Table S2. While *BnC6FTb* exon III turned out to be highly conserved, exon IV exhibited larger sequence diversity. Within 41 bp exon III of the *BnC6FTb* gene, only a single polymorphism was found at position 2004 which corresponds to an allele frequency of 1%. The EMS mutation G1968A resides within a sequence domain which is monomorphic among all accessions investigated. For *BnC6FTb* (exon IV), six polymorphic regions were found with minor allele frequencies of <5.0%. The EMS-generated alleles (positions G2122, G2133, and G2154) are residing in monomorphic sequences (Supplementary Figure 3).

In contrast to *BnFT* genes, a higher variability in *BnTFL1-2* exon III than in exon IV was found. In exon III the SNP showing the largest variation was a C insertion at position 731. The EMS mutation G750A which is also located within this exon, was located in a fully monomorphic domain. For *BnTFL1-2* exon IV, only a T/C polymorphism was found at position 1030. In conclusion, there is a high degree of sequence conservation within the analyzed sequences. Our EMS treatment created novel sequence variations within these highly conserved regions (Supplementary Figure 4). FASTA-formatted sequences for each gene are deposited in Supplementary File 1. All sequences have been submitted to NCBI (www.ncbi.nlm.nih.gov) (accession numbers KJ533546 - KJ533625 and KJ533626 - KJ533728).

### A *BnC6FTb*-splice-site mutation impacts the expression of other flowering time genes in leaves

We reasoned that a loss of function of the *BnC6FTb* paralog directly impacts the transcriptional activity of other major flowering time regulators downstream of *BnFT*. To test this hypothesis, we selected the *BnC6FTb*_*G*2009*A*_ mutant because, first the G2009A SNP causes a splice-site mutation that leads to a truncated protein, and second, *C6FTb*_*G*2009*A*_ M_3_ plants are characterized by a marked flowering delay of about 29 days compared to the Express 617 control (Figure 3).

We chose *BnAP1* and *BnSOC1* as putative downstream targets of *BnFT* genes based on our knowledge from Arabidopsis (Yoo et al., 2005; Kaufmann et al., 2010). We measured their expression in leaves. *BnC6FTb*_*G*2009*A*_ M_3_ plants were grown in the greenhouse under constant temperature and LD conditions. For expression analysis, young leaves of three different plants were taken at stages BBCH30 (pre and post-vernalization), BBCH50, and BBCH60. Arabidopsis *AP1* and *SOC1* sequences were BLASTed against *B. oleracea* and *B. rapa*. High homology hits were aligned and primers were designed from highly conserved regions. Subsequently, joint expression of all paralogs was measured by RT-qPCR. Gene expression levels of *BnAP1* and *BnSOC1* were normalized using *BnGAPDH* and *BnB-Tub* genes.

**Figure 5:**
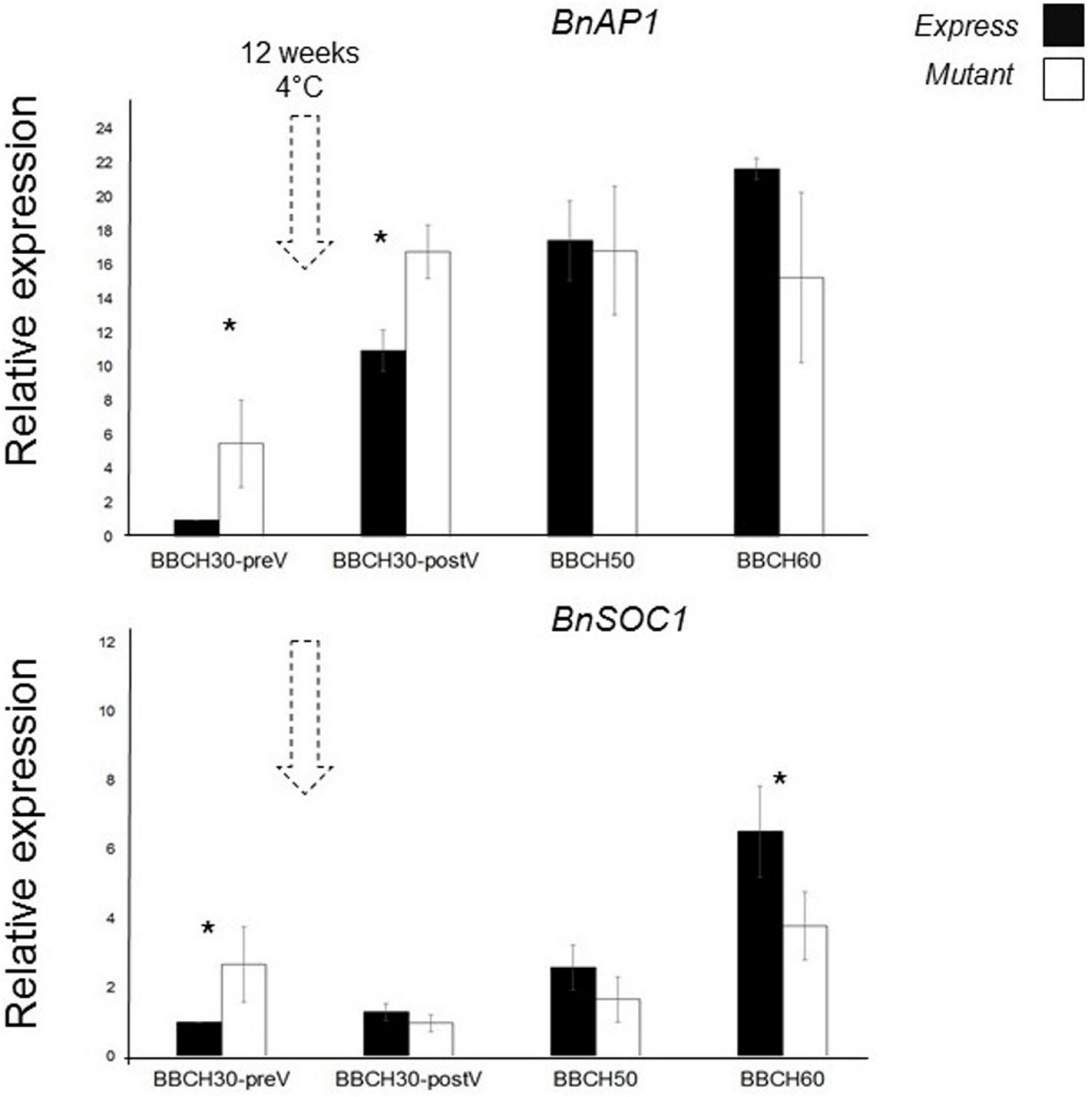
**Joined expression analysis of two *BnFT* downstream targets in the *BnC6FTb*_*G*2009_ mutant (open boxes) and Express 617 (filled boxes)**. Four developmental stages were analyzed before and after vernalization (dotted arrows). Plants at BBCH30 were analyzed before vernalization (preV) and after vernalization (postV). Two biological replicates (M_3_ plants) and three technical repetitions were analyzed for each time point. Error bars: standard error of the mean for biological replicates. Ct of target genes were normalized against the *BnGAPDH* and *BnB-Tub* total expression. The time point BBCH30-preV in Express 617 control plants was taken as reference sample for calculation of relative expression. Differences in relative expression were pairwise tested (control Vs M_3_ line) via *t*-test. Significant differences (*P* < 0.05) are depicted by asterisks. All samples were taken between zeitgeber 11 h and 12 h.

We detected altered transcriptional activities of *BnAP1* in *BnC6FTb*_*G*2009*A*_ mutants when compared to Express 617 control plants. Control Express 617 plants at BBCH30 (preV) were used as reference sample for relative expression calculations. At rosette stages (BBCH30), *BnAP1* expression in the M_3_ mutantwas higher than in control plants, while at BBCH50 and BBCH60 relative expression levels were 10–40% lower (Figure 5). Before vernalization (BBCH30-preV), *BnSOC1* expression in mutants was 2.5-fold higher than in Express 617. After vernalization, we detected a reduction of *BnSOC1* in *BnC6FTb*_*G*2009*A*_ mutants compared to the control. When the first flower opened, the difference in expression between *BnC6FTb*_*G*2009*A*_ mutants and Express 617 was at its maximum. The altered expression in leaves indicates that a single *BnC6FTb* mutation may affect other major flowering time regulators. We expect that both genes are expressed in the shoot apical meristem as well.

### Performance of F_1_ hybrids using the *BnTFL1-2* mutants as parents

In tomato, mutations in *FT* and *TFL1* orthologs accounted for fruit yield heterosis in F_1_ hybrids (Krieger et al., 2010). We made an initial experiment to address the question whether *B. napus* orthologs might have a similar function. For producing F_1_ hybrids, we selected *BnC6FTb*_*G*2009*A*_ and *BnTFL1-2*_*G*750*A*_ homozygous M_3_ mutants as pollinators due to their late flowering phenotype in conjunction with an altered inflorescence (lower number of fertile flowers, Supplementary Figure 1). We crossed homozygous M_3_ plants with the male-sterile (MS) line MSL007. The MSL007 line (MSL-Express) is an isogenic line of Express that carries the male sterility Lembke (MSL) genic male sterility system (Basunanda et al., 2010). Thus, no F_1_ heterosis was expected, except effects due to EMS mutations.

**Figure 6:**
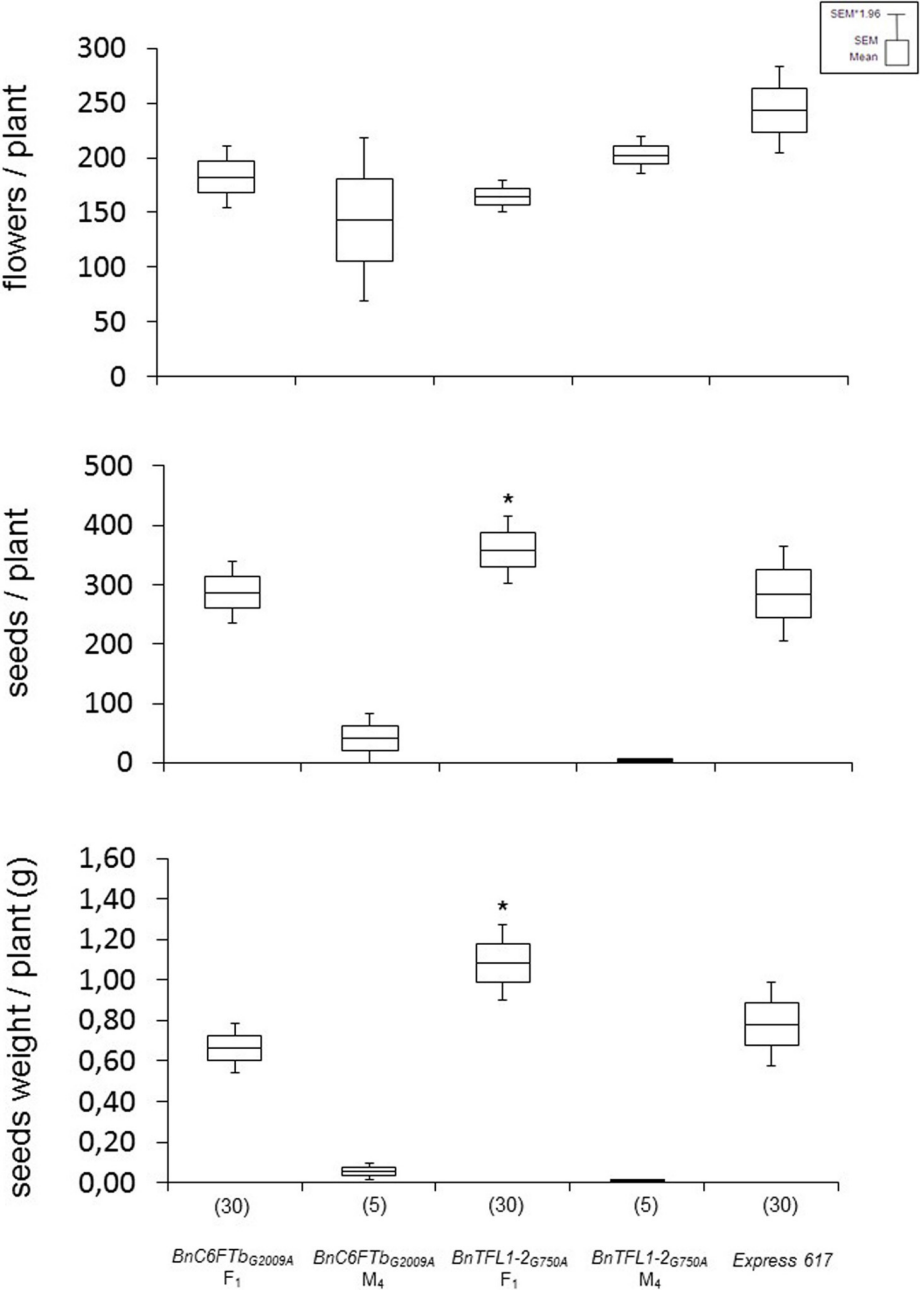
**Growth experiments with vernalized F_1_ hybrids after crossing two mutants with a male sterile isogenic line (MSL007)**. Each F_1_ was obtained from two different crossing experiments. Mutant parents (M_4_ lines), and Express 617 were used as controls. Yield components were determined on single plants grown in the greenhouse. The number of plants analyzed is given in brackets. Differences in flowers, seed number and seed weight per plant against Express 617 were tested via *t*-test. Significant differences (*P* < 0.05) are depicted by asterisks. Growth conditions: 22°C, 16 h light, greenhouse. Vernalization: 4°C, 16 h light, 8 weeks.

F_1_ hybrids were vernalized and grown in the greenhouse with the parental lines and Express 617. Both F_1_ hybrids showed differential effects. *BnC6FTb*_*G*2009*A*_ hybrids showed no significant differences in seed number per plant and total seed weight in comparison to Express 617 as the best parent. In contrast, *BnTFL1-2*_*G*750*A*_ F_1_ hybrids had significantly higher number of seeds/plant (20%) and total seed weight (40%) as compared to the best parent (Figure 6). Although the effects of background mutations cannot be ruled out, these results could indicate that *BnTFL1* mutations impact heterosis in *B. napus*.

## Discussion

In the present study, more than 100 EMS-alleles have been found for three flowering time genes of B. napus. Based on previous reports, the average EMS-mutation frequencies are expected to be lower in diploid species (~1/380 kb) than in polyploids (~1/50 kb) (Till et al., 2007; Wang et al., 2012c). In this study, mutation frequencies ranged between 1/24 kb and 1/72 kb. Using the same EMS-population, mutation frequencies ranged between 1/12 and 1/22 kb for sinapine biosynthesis genes (Harloff et al., 2012). Although the observed *BnFT /BnTFL1* mutation frequencies are slightly lower, our results are in the range expected for polyploid species. The present mutants will be a valuable resource to study flowering regulatory networks in polyploids and they can be introduced into breeding programs.

Our aim was to provide data about the function of *FT* and *TFL1* paralogs in rapeseed. We found that, despite the redundancy of mutations in a single gene, either non-sense or missense mutations in the *BnC6FTb* gene resulted in a marked flowering delay. This supports our hypothesis that *BnFT* paralogs contribute differently to flowering time regulation. A large plethora of Arabidopsis reports on *FT* loss-of-function mutants have established a robust correlation between *FT* mutations and flowering time delay in Arabidopsis (Andres and Coupland, 2012). Contrasting with expectations based on Arabidopsis *TFL1*-phenotypes, *BnTFL1-2* mutants showed a slight delay in flowering time. In Arabidopsis, a single amino acid change in *TFL1* (*tfl1-1*_*Gly*105*Asp*_) led to early flowering and limited the development of indeterminate inflorescence by promoting the formation of a terminal floral meristem (Bradley et al., 1997). On the other hand, F_1_ hybrids derived from crosses between *BnTFL1-2* M_3_- and rapeseed MS lines showed increased seed yield compared to *BnC6FTb* F_1_ hybrids and Express 617 controls. Thus, although the role of *BnTFL1-2* involving flowering time regulation is not likely to be conserved compared to its Arabidopsis ortholog, *TFL1-2* appears to be involved in yield-related traits as reported for its tomato ortholog *SP* (Jiang et al., 2013b). As we mention in the following sections, confirming this hypothesis is a must for new research approaches.

During evolution, duplicated genes may undergo dosage adjustments (Papp et al., 2003; Conant and Wolfe, 2008), non-functionalization, or sub-/neo-functionalization (Force et al., 1999). We wanted to know whether different *BnFT* and *BnTFL1* paralogs gained different function by studying their phenotypes and their transcriptional activities. Through digital gene expression analyses, differential expression within early generations of re-synthesized- (F_1_-F_4_) and natural *B. napus* accessions has been reported (Birchler and Veitia, 2010). Three highly similar genes encoding endoplasmic reticulum-bound sn-glycerol-3-phosphate acyltransferase-4 (*BnGPAT4-C1, BnGPAT4-C2*, and *BnGPAT4-A1*) showed different expression patterns and altered epigenetic features (Chen et al., 2011) which is in accordance with the assumption that in polyploids orthologous genes are frequently expressed in a non-additive manner (Jiang et al., 2013a). We have also observed marked differences in the expression of six *BnFT* paralogs in support of the non/sub-functionalisation hypothesis. In regard to their position within a major flowering time QTL (Wang et al., 2009), *BnC6FTb* paralogs seem to play the most important role as flowering time regulators in winter type *B. napus*. More evidence has been given by the expression analysis of putative *FT* downstream targets *BnAP1* and *BnSOC1* in late-flowering *BnC6FTb*_*G*2009*A*_ mutants. *SOC1* encodes a MADS-box transcription factor, acting as a floral integrator (Lee and Lee, 2010). The gene *SOC1* gene is expressed in the shoot apical meristem, and *SOC1* mutations lead to late flowering phenotype (Borner et al., 2000). However, *SOC1* is also expressed in vegetative organs (leaves) (Hepworth et al., 2002). A reduction in *SOC1* mRNA was detected in the meristem of late-flowering *ft-7* (Trp138Stop) *Arabidopsis* Ler background (Searle et al., 2006). In the future, the activities of these genes shall also be studied in the shoot apical meristem.

The phenotypic studies presented here gave further support to our assumption that *BnFT* paralogs do not contribute equally to flowering time regulation. In sugar beet (*Beta vulgaris*), two *FT* paralogs (*BvFT1* and *BvFT2*) were reported to antagonistically regulate flowering time (Pin et al., 2010). Knockdown of the *FT* potato paralog *StSP3D* resulted in a late flowering phenotype, while knockdown of the second paralog *StSP6A* had no effect on flowering time but on tuberization (Navarro et al., 2011). In line with these findings, we observed differential effects of *BnC6FTa* and *BnC6FTb* mutations. The strong effect of *BnC6FTb* as a flowering time regulator in rapeseed has been confirmed. To which extend *BnC6FT*a and other *BnFT* paralogs are involved in flowering time control needs to be investigated in the future (e.g., by using other TILLING mutants). As determined by our expression analyses, the strongest case of non-functionalization is shown by the lack of expression of the *BnC2FT* copy. This result is in full congruence with Wang et al. report (2012a) where this gene copy was neither expressed in *B. napus* nor in *B. oleracea*. A series of recent studies has demonstrated that beyond flowering time control, FT-like proteins act as mobile or cell-autonomous proteins that mediate other developmental processes, such as growth, plant architecture, and tuber formation (Carmona et al., 2007; Kinoshita et al., 2011; Navarro et al., 2011). In contrast to our *BnFT* mutant results, a previous analysis of sinapine biosynthesis mutants from the same EMS population as in our study, phenotypic or physiological effects had been observed only in double mutants (Harloff et al., 2012, Harloff, personal communication).

Although yield heterosis is regarded as a quantitative trait, single genes can contribute to heterotic effects through overdominance, such as the Arabidopsis *Erecta* locus (Moore and Lukens, 2011). As a first example of single gene overdominance, the yield of *sft-4537*/± heterozygous tomato plants was increased by up to 60% in comparison to their parents after crossing high yielding M82 inbred plants with low-yielding homozygous loss of function mutants (*sft-4537*) (Krieger et al., 2010). In our work, F_1_ hybrids carrying a *BnTFL1-2* mutant allele had a higher seed yield as the Express 617 parent. Our study delivers the first insights about potential *TFL1*-related heterosis in *B. napus*. In the future, experimental data are needed to verify this hypothesis. We tested our initial hypothesis by crossing mutants with the non-mutated donor line. Although, the data point at *BnTFL1* as a major gene for heterosis in rapeseed the possible impact of background mutations must be analyzed by additional hybrid combinations. Moreover, we will sequence the *BnTFL1-2* loci from rapeseed lines with high and low combining ability (Qian et al., 2007). If our preliminary greenhouse data will be confirmed by field experiments, this study will offer new perspectives for a hybrid breeding strategy which make use of *BnTFL1-2* sequence variations.

### Conflict of interest statement

The authors declare that the research was conducted in the absence of any commercial or financial relationships that could be construed as a potential conflict of interest.
