# Supplementary material for: Mutations in single FT- and TFL1-paralogs of rapeseed (Brassica napus L.) and their impact on flowering time and yield components
Source: Front Plant Sci. 2014 Jun 17;5:282. doi: 10.3389/fpls.2014.00282 (PMC4060206; doi:10.3389/fpls.2014.00282)
Supplement: Supplementary file 1 [file DataSheet1.DOCX]

***Supplementary Material***

**Mutations in single *FT-* and *TFL1-*paralogs of rapeseed (*Brassica napus* L.) and their impact on flowering time and yield components**

**Guo, Yuan^1^, Harloff Hans^1^, Jung Christian^1^ , Carlos Molina^*^**

^1^Plant Breeding Institute, Kiel University, Olshausenstrasse 40, D-24098 Kiel, Germany

*** Correspondence:** Carlos Molina, Plant Breeding Institute, Kiel University, Olshausenstrasse 40, D-24098 Kiel, Germany

c.molina@plantbreeding.uni-kiel.de

1. **Supplementary Data**

**Note: Supplementary files are uploaded as ZIP file along with the main manuscript.**

Multi FASTA file containing sequences *BnC6FTb* and *BnTFL1-2* from a subset of the *Brassica napus ASSYST* diversity set:

*ASSYST subset BnC6FTb-BnTFL1-2.txt*

1. **Supplementary Figures and Tables**

## Supplementary Tables

**Supplementary Table S1** PCR primers used in this study for screening EMS-mutations and for expression analysis by RT-qPCR

| Target gene | Primer name | Orientation | Sequence (5’-3’) | Application |
| --- | --- | --- | --- | --- |
| *BnC6FTb-exon III-IV* | *BnC6FTb_F9* | Forward | GCTACACACTAAGCCTATTTGTAT | TILLING |
| *BnC6FTb-exon III-IV* | *BnC6FTb_R12* | Reverse | CCACCCTGGTTCATACACC | TILLING |
| *BnC6FTb-exon I* | *BnC6FTb_P1* | Forward | ATTTTGAGTCACAAGTCGC | TILLING |
| *BnC6FTb-exon I* | *BnC6FTb_R1* | Reverse | CGGTAGGTTGTAGCTACATGTA | TILLING |
| *BnC6FTb-exon I* | *BnC6FTb_P4-nest* | Forward | CTCGTACATCACTGTATACAGG | TILLING |
| *BnC6FTb-exon I* | *BnC6FTb_R1-nest* | Reverse | CGGTAGGTTGTAGCTACATGTA | TILLING |
| *BnC6FTa-exon I* | *BnC6FTa_P1* | Forward | TTCTAACACAGATTTCGCTG | TILLING |
| *BnC6FTa-exon I* | *BnC6FTa_R1* | Reverse | CGGTAGGTTGTAGCTACGTG | TILLING |
| *BnC6FTa-exon* | *BnC6FTa_P2-nest* | Forward | TCTCGTCCATCACTGTACAGA | TILLING |
| *BnC6FTa-exon I* | *BnC6FTa_R1-nest* | Reverse | CGGTAGGTTGTAGCTACGTG | TILLING |
| *BnTFL1-2* | *BnTFL1_F1* | Forward | TCTCTCTTTCCCTAAGCTCACTT | TILLING |
| *BnTFL1-2* | *BnTFL1_2_R1* | Reverse | CGGGAATTTAATCTCCTATAA TGAG | TILLING |
| *BnGAPDH* | *BnGADPH-3_Fw* | Forward | CCGCTTCCTTCAACATCATT | RT-qPCR |
| *BnGAPDH* | *BnGADPH-3_Rev* | Reverse | GTCGCAGCTTTCTCGAGTCT | RT-qPCR |
| *BnB-Tub* | *BnB-Tub_Fw* | Forward | CAGATGTGGGATGCAAAGAA | RT-qPCR |
| *BnB-Tub* | *BnB-Tub_Rev* | Reverse | TCCATTCCACAAAGTAGGATGA | RT-qPCR |
| *BnAP1* | *BnAP1_F3* | Forward | GGCGAGAGAGAAGATAAGGATA | RT-qPCR |
| *BnAP1* | *BnAP1_R1* | Reverse | GCTAGAGAACTCGAAGAGCTTTCC | RT-qPCR |
| *BnSOC1* | *BnSOC1_F1* | Forward | GCTGCAGAAAACCAGAAGCTCACTG | RT-qPCR |
| *BnSOC1* | *BnSOC1_R2* | Reverse | TGAAGAAGAAGGCAACCCAATG | RT-qPCR |
| *BnC6FTb* | *BnC6FTb_Fw* | Forward | AATGAGATTGTGTCTTACGAGAA | RT-qPCR |
| *BnC6FTb* | *BnC6FTb_Rev* | Reverse | AAGGCCGAGATTGTATAGCGCA | RT-qPCR |
| *BnA7FTb* | *BnA7FTb_Fw* | Forward | AATGAGATTGTGTCTTACGAGAA | RT-qPCR |
| *BnA7FTb* | *BnA7FTb_Rev* | Reverse | AAGGCCGAGATTGTATAGCGCG | RT-qPCR |
| *BnC6FTa* | *BnC6FTa_Fw* | Forward | CCCACCTCGGGAATTCATCGTC | RT-qPCR |
| *BnC6FTa* | *BnC6FTa_Rev* | Reverse | TAAACCGCAGCCACGGGAAGGCT | RT-qPCR |
| *BnA7FTa* | *BnA7FTa_Fw* | Forward | CCCACCTCGGGAATTCATCGTC | RT-qPCR |
| *BnA7FTa* | *BnA7FTa_Rev* | Reverse | TAAACCGCAGCCACGGGAAGGCC | RT-qPCR |
| *BnA2FT* | *BnA2_Fw* | Forward | GTTGTAGGAGACGTTCTTGAATGT | RT-qPCR |
| *BnA2FT* | *BnA2_Rev* | Reverse | TCTGGATCCACCATAACCAAAGTA | RT-qPCR |
| *BnC2FT* | *BnC2_Fw* | Forward | GTTGTAGGAGACGTTCTTGAATGT | RT-qPCR |
| *BnC2FT* | *BnC2_Rev* | Reverse | TCTGGATCCACCATAACCAAAGTG | RT-qPCR |

**Supplementary Table S2** *B. napus* accessions selected for *BnC6FTb* and *BnTFL1-1* sequencing. Plant materials are classified into four growth types: winter (W), spring (S), semi-winter (SW), and swede (SD).

| ASSYST Number | Accession Name | Type |  | ASSYST Number | Accession Name | Type |
| --- | --- | --- | --- | --- | --- | --- |
| ASSYST-001 | Alesi | W |  | ASSYST-080 | Orlando | W |
| ASSYST-002 | Remy | W |  | ASSYST-086 | Lilian | W |
| ASSYST-003 | Robust | W |  | ASSYST-088 | KW1519 | W |
| ASSYST-004 | Alaska | W |  | ASSYST-089 | AMBER x COMANCHE | W |
| ASSYST-005 | Pirola | W |  | ASSYST-096 | HANSEN x GASPARD DH LINE | W |
| ASSYST-006 | Adder | W |  | ASSYST-098 | RAFAL DH1 | W |
| ASSYST-007 | Milena | W |  | ASSYST-102 | Lesira | W |
| ASSYST-008 | Allure | W |  | ASSYST-113 | Samourai | W |
| ASSYST-009 | Agalon | W |  | ASSYST-117 | Maplus | W |
| ASSYST-011 | Picasso | W |  | ASSYST-119 | Lirabon | W |
| ASSYST-012 | Lord | W |  | ASSYST-128 | Leopard | W |
| ASSYST-013 | KW3077 | W |  | ASSYST-130 | Resyn-Gö S4 | W |
| ASSYST-014 | Rodeo | W |  | ASSYST-131 | Resyn-Gö H226 | W |
| ASSYST-015 | Rapid | W |  | ASSYST-150 | Kromerska | W |
| ASSYST-016 | Boston | W |  | ASSYST-160 | Matador | W |
| ASSYST-017 | Escort | W |  | ASSYST-166 | Panter | W |
| ASSYST-018 | Montego | W |  | ASSYST-178 | Vinnickij 15/59 | W |
| ASSYST-021 | Savannah | W |  | ASSYST-180 | V8 | W |
| ASSYST-024 | Ladoga | W |  | ASSYST-181 | 1012-98 | W |
| ASSYST-026 | Cooper | W |  | ASSYST-185 | Canard | W |
| ASSYST-034 | Lipid | W |  | ASSYST-193 | Dwarf Essex | W |
| ASSYST-046 | Smart | W |  | ASSYST-197 | Nunsdale | W |
| ASSYST-049 | NK Fair | W |  | ASSYST-210 | RED RUSSIAN | W |
| ASSYST-052 | SWGospel | W |  | ASSYST-212 | SLAPSKA, SLAPY | W |
| ASSYST-054 | Tenor | W |  | ASSYST-218 | GROENE GRONINGER SNIJMOES | W |
| ASSYST-055 | Expert | W |  | ASSYST-230 | SWU Chinese 2 | SW |
| ASSYST-059 | SW Sinatra | W |  | ASSYST-232 | SWU Chinese 5 | SW |
| ASSYST-067 | Nugget | W |  | ASSYST-236 | SWU Chinese 9 | SW |

**Supplementary Table S2 (Continuation)**

| ASSYST Number | Accession Name | Type |  | ASSYST Number | Accession Name | Type |
| --- | --- | --- | --- | --- | --- | --- |
| ASSYST-237 | Zhouyou | SW |  | ASSYST-312 | Adamo | S |
| ASSYST-239 | STELLAR DH | S |  | ASSYST-313 | Altex | S |
| ASSYST-240 | WESTAR DH10 | S |  | ASSYST-314 | Andor | S |
| ASSYST-241 | YUDAL | S |  | ASSYST-315 | Astor | S |
| ASSYST-242 | BRUTOR | S |  | ASSYST-316 | Aurora | S |
| ASSYST-244 | COMET | S |  | ASSYST-317 | Bingo | S |
| ASSYST-250 | INDUSTRY | S |  | ASSYST-318 | Callypso | S |
| ASSYST-251 | KARAT | S |  | ASSYST-325 | Erake | S |
| ASSYST-253 | NIKLAS | S |  | ASSYST-327 | Futura | S |
| ASSYST-254 | TARGET | S |  | ASSYST-328 | Galant | S |
| ASSYST-256 | KAROO-057DH | S |  | ASSYST-329 | Giant Xr707 | S |
| ASSYST-268 | MAZOWIECKI | S |  | ASSYST-330 | Gisora | S |
| ASSYST-271 | Liho | S |  | ASSYST-334 | Gulliver | S |
| ASSYST-272 | Alku | S |  | ASSYST-335 | Hankkija's Lauri | S |
| ASSYST-274 | Ceska Krajova | S |  | ASSYST-339 | Korall | S |
| ASSYST-276 | Janetzkis Sommerraps | S |  | ASSYST-340 | Korinth | S |
| ASSYST-278 | Marnoo | S |  | ASSYST-342 | Kruglik | S |
| ASSYST-280 | Olga | S |  | ASSYST-346 | Liraspa | S |
| ASSYST-281 | Spaeths Zollerngold | S |  | ASSYST-347 | Lirawell | S |
| ASSYST-284 | Tribute | S |  | ASSYST-348 | Lisandra | S |
| ASSYST-285 | Wesway | S |  | ASSYST-349 | Loras | S |
| ASSYST-288 | Tower | S |  | ASSYST-360 | Olivia | S |
| ASSYST-290 | Ability | S |  | ASSYST-361 | Omega | S |
| ASSYST-291 | Campino | S |  | ASSYST-366 | Pivot | S |
| ASSYST-299 | Larissa | S |  | ASSYST-372 | Rsio | S |
| ASSYST-300 | Magma | S |  | ASSYST-374 | Sabine | S |
| ASSYST-302 | Mozart | S |  | ASSYST-427 | Essex Model | SD |
| ASSYST-305 | Pauline | S |  | ASSYST-431 | Peerless (Acme) | SD |
| ASSYST-306 | Sophia | S |  | ASSYST-454 | Troendersk Kvithamar | SD |
| ASSYST-307 | Tribune | S |  | ASSYST-466 | Ruta Otofte | SD |
| ASSYST-309 | Rivette | S |  |  |  |  |

**Supplementary Table S3** M_3_ families originating mutations in three flowering time genes and their segregation into three genotypic classes (*FT FT*: wild-type.; *FT ft*: heterozygous; *ft ft*: homozygous mutants). M_3_ seeds had been produced after selfing M_2_ plants of the Express617 EMS population.

| **Gene** | **Mutation** | ***FT FT*** | ***FT ft*** |  | ***ft/ft*** | **Chi^2^ Value** | **P-Value** | **Seed code** |
| --- | --- | --- | --- | --- | --- | --- | --- | --- |
| *BnC6FTb* | *C6FTb_G2154A_* | 0 | 0 |  | 16 |  |  | 110104 |
| *BnC6FTb* | *C6FTb_G2122A_* | 0 | 0 |  | 29 |  |  | 114619 |
| *BnC6FTb* | *C6FTb_G2009A_* | 0 | 0 |  | 28 |  |  | 114620 |
| *BnC6FTb* | *C6FTb_C2133T_* | 4 | 8 |  | 8 | 2.400 | 0.3012 | 110103 |
| *BnC6FTb* | *C6FTb_C666T_* | 5 | 8 |  | 5 | 0.222 | 0.8948 | 110106 |
| *BnC6FTb* | *C6FTb_G17A_* | 0 | 5 |  | 6 | 6.636 | 0.0362 | 110109 |
| *BnC6FTb* | *C6FTb_G124A_* | 6 | 8 |  | 3 | 1.118 | 0.5719 | 110110 |
| *BnC6FTb* | *C6FTb_G1968A_* | 2 | 15 |  | 5 | 0.155 | 0.5719 | 114623 |
| *BnC6FTa* | *C6FTa_G163A_* | 0 | 0 |  | 25 |  |  | 114614 |
| *BnC6FTa* | *C6FTa_G104A_* | 0 | 0 |  | 7 |  |  | 114615 |
| *BnC6FTa* | *C6FTa_G37A_* | 0 | 0 |  | 18 |  |  | 114617 |
| *BnC6FTa* | *C6FTa_G52A_* | 5 | 10 |  | 3 | 0.667 | 0.717 | 114612 |
| *BnC6FTa* | *C6FTa_C74T_* | 2 | 13 |  | 5 | 2.700 | 0.259 | 114613 |
| *BnTFL1-2* | *TFL1_G750A_* | 0 | 0 |  | 19 |  |  | 114640 |
| *BnTFL1-2* | *TFL1_G52A_* | 0 | 0 |  | 24 |  |  | 114641 |
| *BnTFL1-2* | *TFL1_G851A_* | 5 | 13 |  | 3 | 1.571 | 0.456 | 114629 |
| *BnTFL1-2* | *TFL1_C518T_* | 5 | 12 |  | 8 | 0.760 | 0.684 | 114633 |
| *BnTFL1-2* | *TFL1_C965T_* | 8 | 10 |  | 6 | 1.000 | 0.607 | 114637 |

## Suplementary Figures

**Supplementary Figure 1** Traits performance of eighteen *BnC6FT*- and *BnTFL1-2* mutants grown in the greenhouse at constant temperature (22°C), and LD (16h light) after vernalization (4°C, 16h light, 8 weeks). Total flowers per plant (A) and total filled pods per plant (B) was measured in M_3_ plants homozygous for the EMS allele. The non-mutagenized donor line Express 617 was used as a control. The number of plants analyzed is written in brackets. Differences between homozygous mutants and control plants was tested via t-test. Significant differences (P<0.05) are depicted by asterisks.

**Supplementary Figure 2** Flowering time point of three genotypes (homozygous mutants *ft ft*, heterozygous *FT ft*, wild-type *FT FT*) from the *BnC6FTb_G2154A_* F_2_ population compared to non-mutagenized Express 617 plants. All plants were grown in the greenhouse at constant temperature (22°C) and LD (16h light) after vernalization (4°C, 16h light, 8 weeks). Days to flowering (BBCH 60) was measured for each individual plant. The number of plants analyzed is written in brackets. Differences in flowering time between homozygous mutants and control plants was tested via t-test. Significant differences (P<0.05) are depicted by asterisks.

**Supplementary Figure 3** Sequence analysis of two exons of the *BnC6FTb* paralog from *B. napus*. One-hundred and seventeen oilseed rape cultivars from a collection of 500 accessions of different origins and growth types were sequenced. For *BnC6FTb*, 103 sequences with optimal length were further selected for further analysis Nucleotide polymorphisms are marked in red. Positions of EMS-mutations in Express 617 mutants (sequence not shown) are depicted by arrows.

**Supplementary Figure 4** Sequence analysis of two exons of the *BnTFL1-2* paralog in *B. napus*. One-hundred and seventeen oilseed rape cultivars from a collection of 500 accessions of different origins and growth types were sequenced. For the *BnTFL1-2,* 80 sequences with optimal length were further selected for further analysis. Nucleotide polymorphisms are marked in red. Positions of EMS-mutations in the Express 617 mutants (sequences not shown) are depicted by arrows.

**Supplementary Figure 5** Traits performance of eighteen *BnC6FT*- and *BnTFL1-2* mutants grown in the greenhouse at constant temperature (22°C), and LD (16h light) after vernalization (4°C, 16h light, 8 weeks). Plant height (A) and plant dry weight (B) was measured in M_3_ plants homozygous for the EMS allele. The non-mutagenized donor line Express 617 was used as a control. The number of plants analyzed is written in brackets. Differences between homozygous mutants and control plants was tested via t-test. Significant differences (P<0.05) are depicted by asterisks.

**Supplementary Figure 6** Traits performance of eighteen *BnC6FT*- and *BnTFL1-2* mutants grown in the greenhouse at constant temperature (22°C), and LD (16h light) after vernalization (4°C, 16h light, 8 weeks). Total seed per plant (A) and seed weight per plant (B) was measured in M_3_ plants homozygous for the EMS allele. The non-mutagenized donor line Express 617 was used as a control. The number of plants analyzed is written in brackets. Differences between homozygous mutants and control plants was tested via t-test. Significant differences (P<0.05) are depicted by asterisks.
