# Supplementary figures and images for: Mutations in single FT- and TFL1-paralogs of rapeseed (Brassica napus L.) and their impact on flowering time and yield components
Source: Front Plant Sci. 2014 Jun 17;5:282. doi: 10.3389/fpls.2014.00282 (PMC4060206; doi:10.3389/fpls.2014.00282)

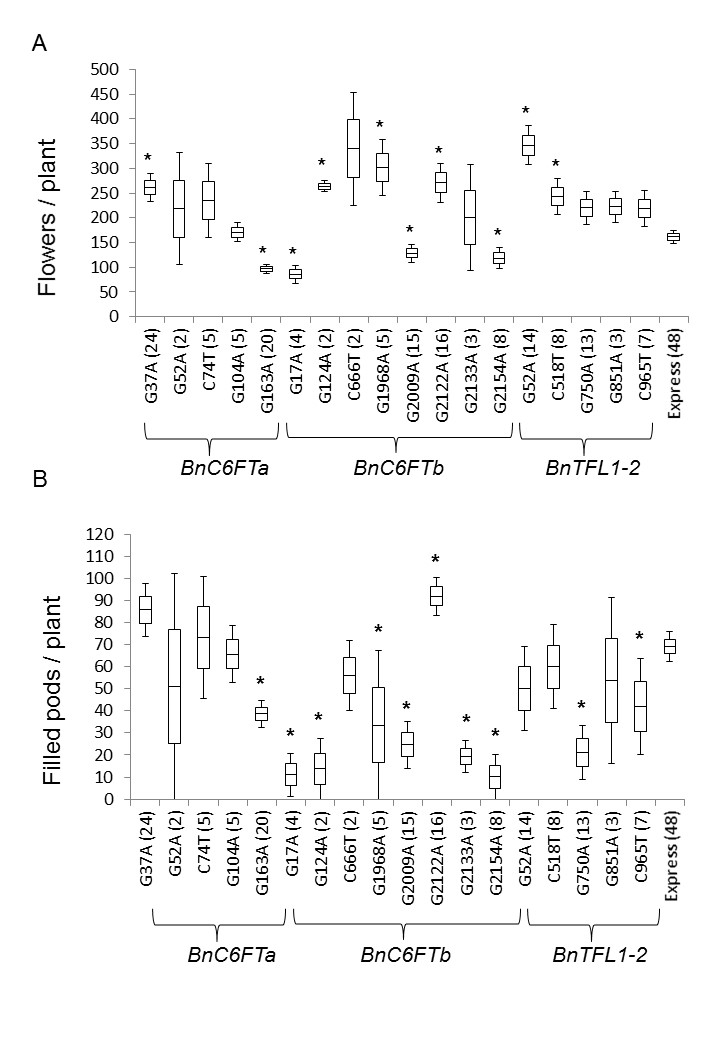

Supplement: Supplementary file 2 [file Presentation1.ZIP › JPEG Supplementary files guo et al/.picasaoriginals/Supplementary figure 1.JPG]

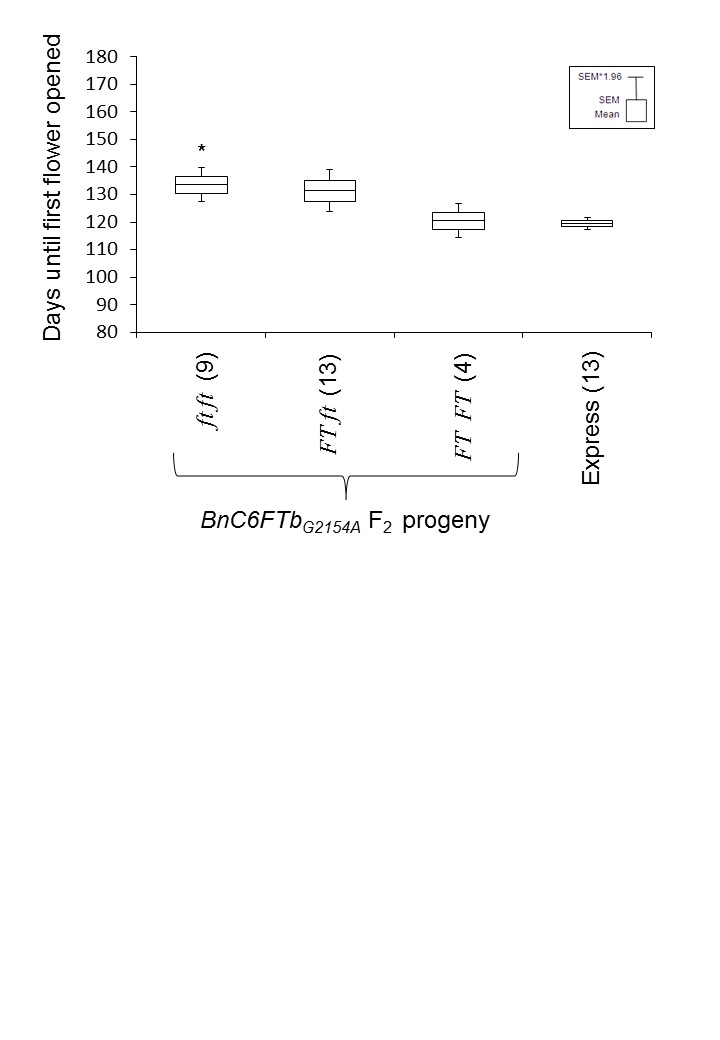

Supplement: Supplementary file 2 [file Presentation1.ZIP › JPEG Supplementary files guo et al/.picasaoriginals/Supplementary figure 2.JPG]

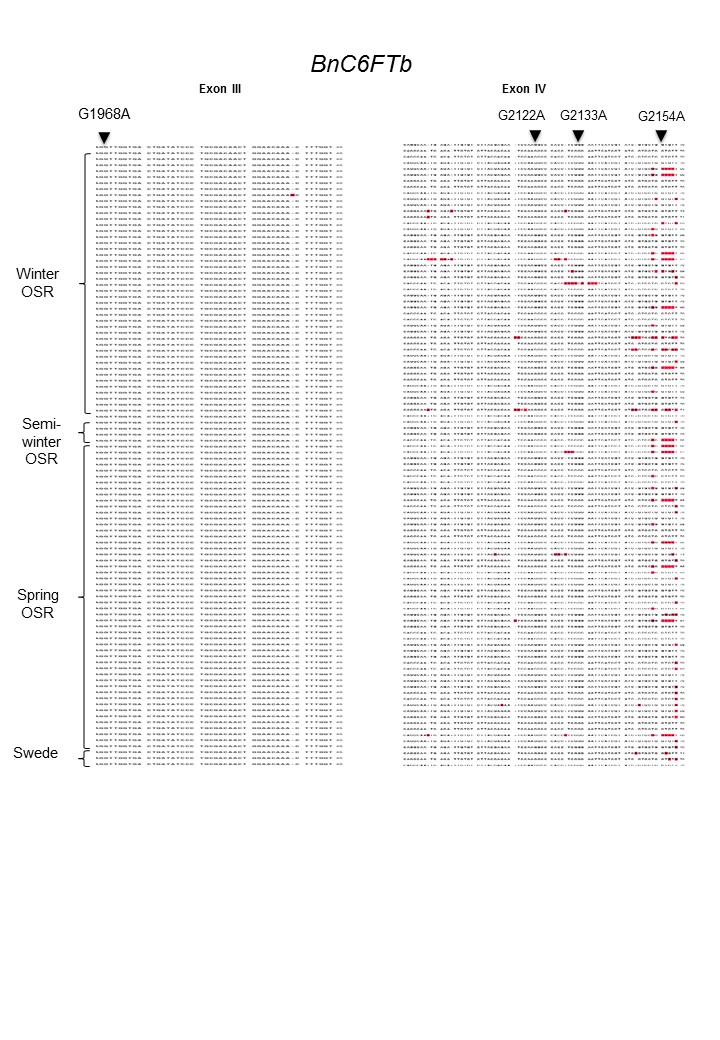

Supplement: Supplementary file 2 [file Presentation1.ZIP › JPEG Supplementary files guo et al/.picasaoriginals/Supplementary figure 3.JPG]

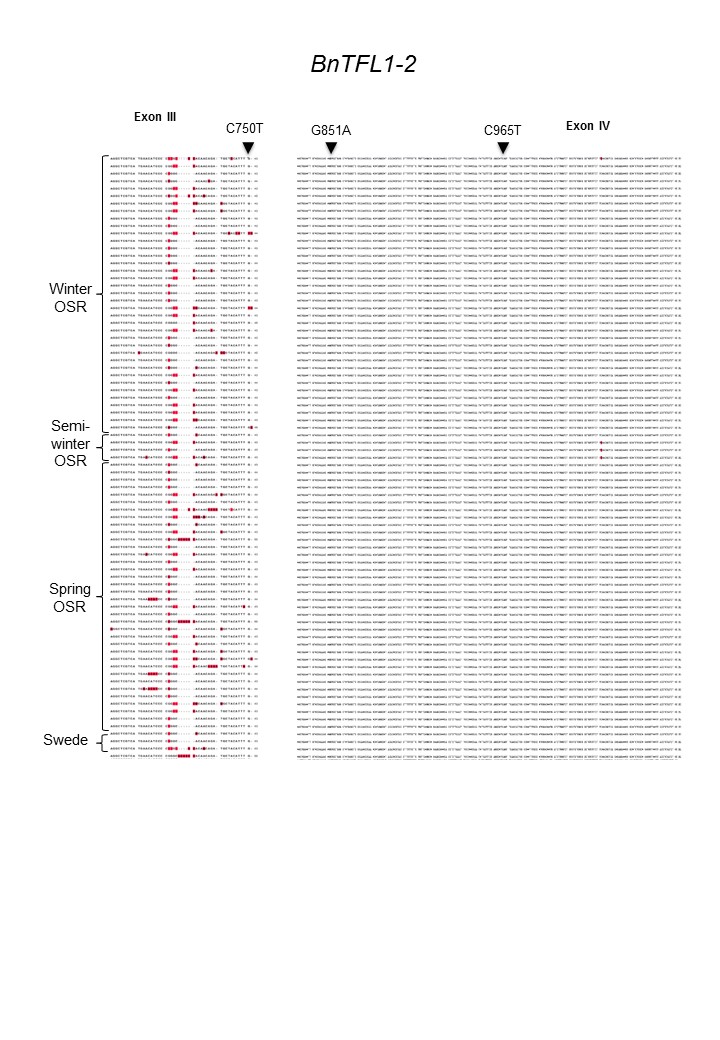

Supplement: Supplementary file 2 [file Presentation1.ZIP › JPEG Supplementary files guo et al/.picasaoriginals/Supplementary figure 4.JPG]

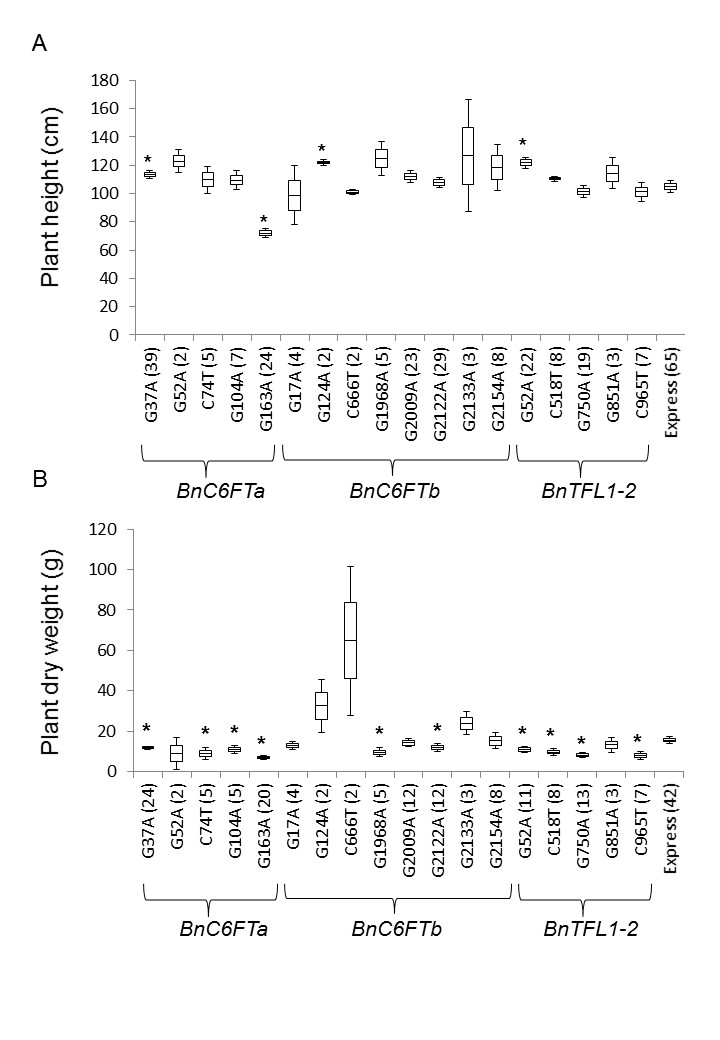

Supplement: Supplementary file 2 [file Presentation1.ZIP › JPEG Supplementary files guo et al/.picasaoriginals/Supplementary figure 5.JPG]

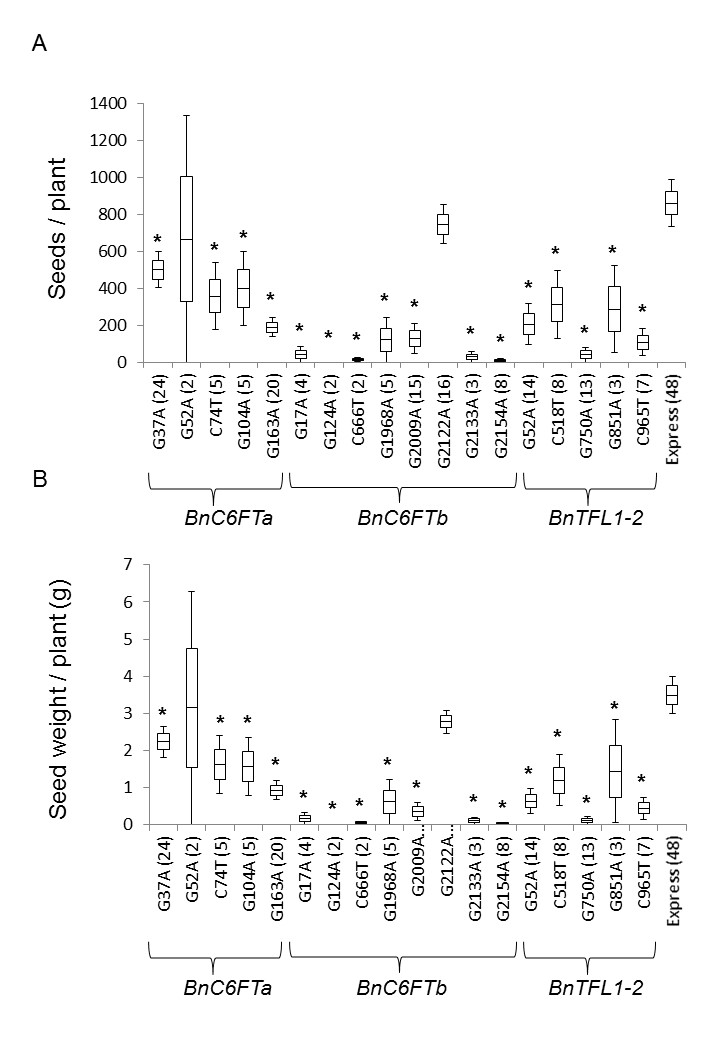

Supplement: Supplementary file 2 [file Presentation1.ZIP › JPEG Supplementary files guo et al/.picasaoriginals/Supplementary figure 6.JPG]

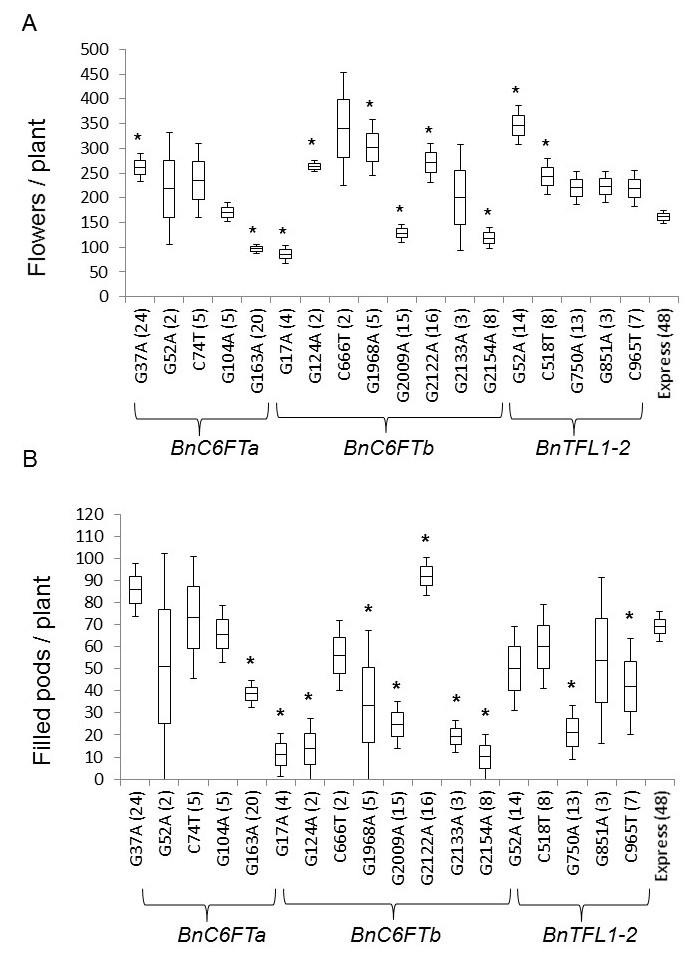

Supplement: Supplementary file 2 [file Presentation1.ZIP › JPEG Supplementary files guo et al/Supplementary figure 1.JPG]

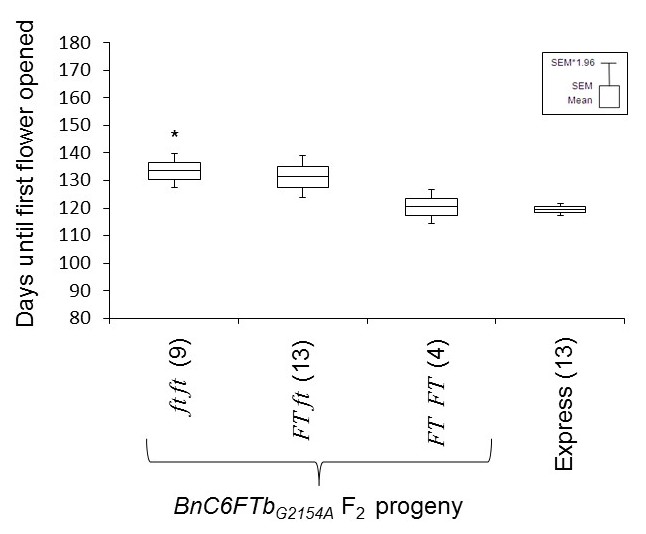

Supplement: Supplementary file 2 [file Presentation1.ZIP › JPEG Supplementary files guo et al/Supplementary figure 2.JPG]

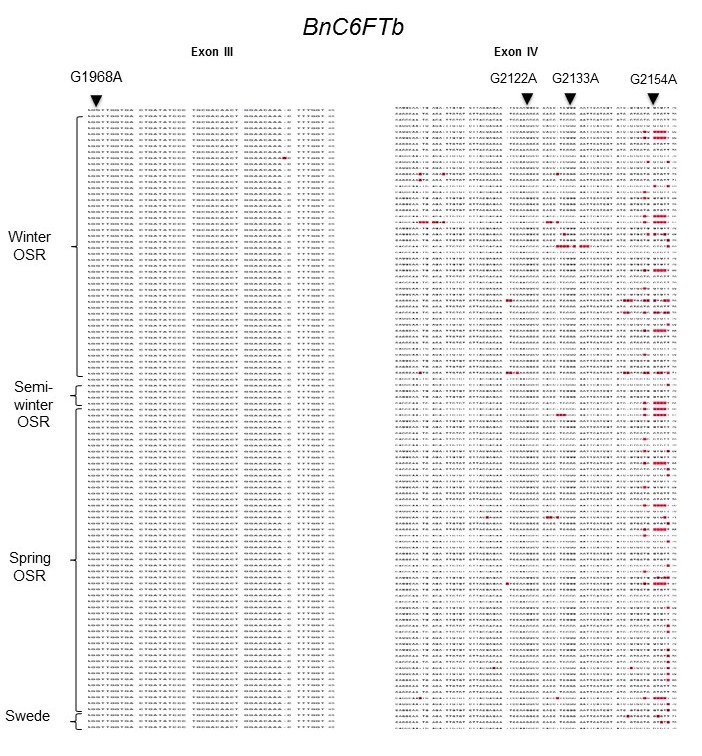

Supplement: Supplementary file 2 [file Presentation1.ZIP › JPEG Supplementary files guo et al/Supplementary figure 3.JPG]

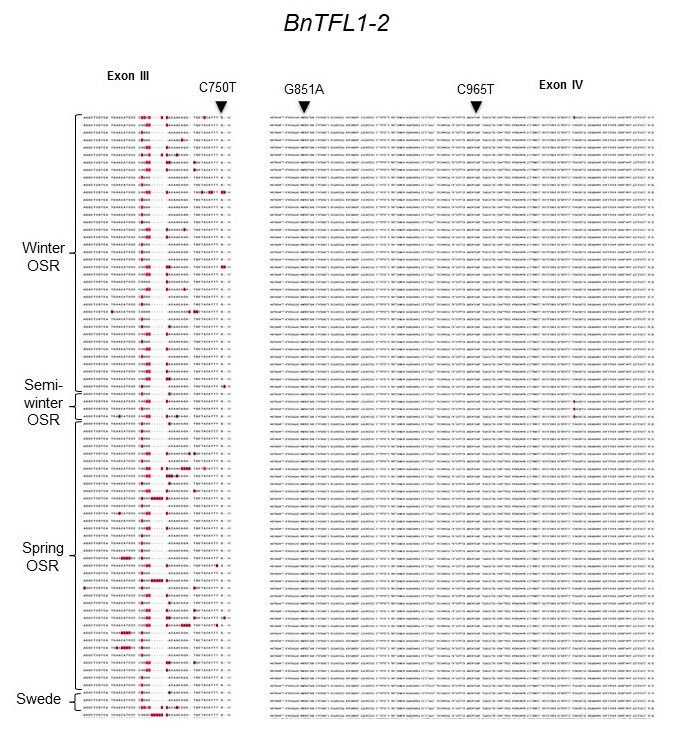

Supplement: Supplementary file 2 [file Presentation1.ZIP › JPEG Supplementary files guo et al/Supplementary figure 4.JPG]

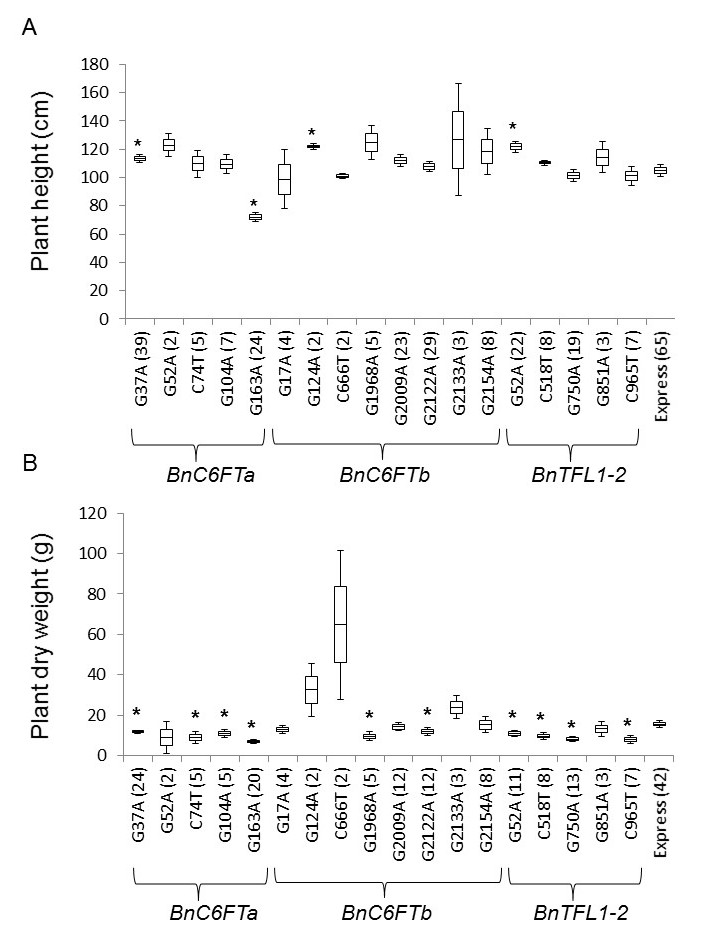

Supplement: Supplementary file 2 [file Presentation1.ZIP › JPEG Supplementary files guo et al/Supplementary figure 5.JPG]

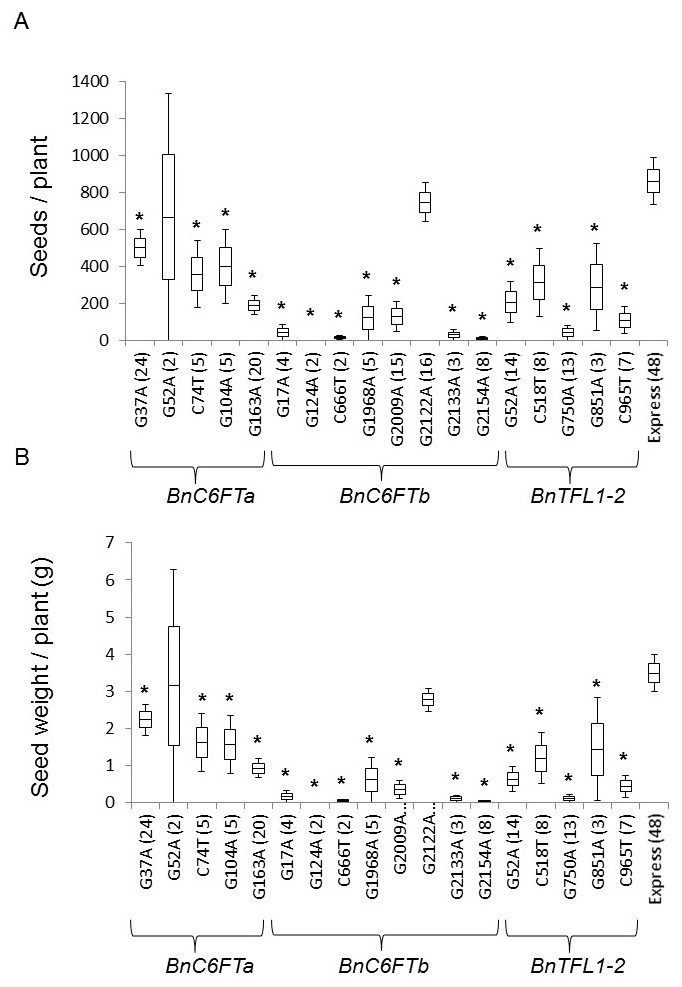

Supplement: Supplementary file 2 [file Presentation1.ZIP › JPEG Supplementary files guo et al/Supplementary figure 6.JPG]
